# Driver gene classification reveals a substantial overrepresentation of tumor suppressors among very large chromatin-regulating proteins

**DOI:** 10.1038/srep38988

**Published:** 2016-12-23

**Authors:** Zeev Waks, Omer Weissbrod, Boaz Carmeli, Raquel Norel, Filippo Utro, Yaara Goldschmidt

**Affiliations:** 1 Machine Learning for Healthcare and Life Sciences, IBM Research – Haifa, Mount Carmel Campus, Israel; 2 Computational Biology Center, IBM T. J. Watson Research, Yorktown Heights, NY 10598, USA

## Abstract

Compiling a comprehensive list of cancer driver genes is imperative for oncology diagnostics and drug development. While driver genes are typically discovered by analysis of tumor genomes, infrequently mutated driver genes often evade detection due to limited sample sizes. Here, we address sample size limitations by integrating tumor genomics data with a wide spectrum of gene-specific properties to search for rare drivers, functionally classify them, and detect features characteristic of driver genes. We show that our approach, *CA*nce*R* ge*N*e similarity-based *A*nnotator and *F*inder (CARNAF), enables detection of potentially novel drivers that eluded over a dozen pan-cancer/multi-tumor type studies. In particular, feature analysis reveals a highly concentrated pool of known and putative tumor suppressors among the <1% of genes that encode very large, chromatin-regulating proteins. Thus, our study highlights the need for deeper characterization of very large, epigenetic regulators in the context of cancer causality.

Cancer originates from a set of genetic aberrations that alter the balance between cell division and death^1^. Genes in which acquired mutations are causally linked to cancer progression are known as drivers. Cancer driver genes can be functionally classified as tumor suppressor genes (TSGs) or oncogenes (OGs) based on their role in disease formation. Unharmed TSGs act to prevent disease onset or progression whereas OGs promote cancer upon acquisition of specific genomic defects.

Numerous genomic and experimental efforts have attempted to refine the compendium of cancer driver genes given their clinical relevance in cancer^2^,^3^,^4^,^5^,^6^,^7^,^8^. However, in spite of immense efforts, evidence suggests the existence of many uncharacterized TSGs and OGs. Perhaps most notably, down-sampling analysis of nearly 5,000 tumor genomes predicted the existence of hundreds of elusive driver genes mutated at intermediate and low frequencies^9^. As mutations do not occur evenly across the genome^10^, mutation frequency is not perfectly correlated with driver gene potency. Thus, infrequently mutated driver genes can potentially have strong phenotypes. In fact, there are sequenced tumors that lack even a single mutation in characterized driver genes^2^,^11^.

Several computational approaches have been employed to detect infrequently mutated, or rare, driver genes. Analysis of mutation patterns rather than frequency circumvents sample size issues to some extent^1^,^12^,^13^,^14^, although drivers with atypical patterns may be missed by such frameworks. Alternatively, dimensionality reduction from genes to gene clusters or pathways can be used to address statistical power limitations, at the cost of bias resulting from incomplete knowledge of protein networks^15^,^16^. Finally, pan-cancer analysis can be used to examine the similarities and differences among the genomic and cellular alterations found across diverse tumor types, thus increasing sample size.

Given the sample size limitations in existing data sets^9^, we hypothesized that gene similarity-based methods may be a promising complementary approach for identifying infrequently mutated drivers. Such statistical methods can create a ranked list of candidate genes by using the vast wealth of available gene-level knowledge to infer statistical patterns that characterize driver genes^17^,^18^,^19^. More importantly, similarity can be used to inform specific traits that can aid in narrowing the driver gene search space. Although several existing methods have also used gene-level knowledge to identify driver genes^15^,^16^,^19^, the collection of gene features used is typically small and does not fully exploit the vast amount of biological knowledge accumulated over the last several years.

In this study, we used a similarity-based machine learning approach and performed driver gene feature analysis using a wide collection of gene properties beyond tumor genomics to detect mutation-based and copy number-based TSGs and OGs. Our classifier, *CA*nce*R* ge*N*e similarity-based *A*nnotator and *F*inder (CARNAF), was used in a pan-cancer mode and identified driver genes which are supported by biomedical literature but were not detected by 15 existing studies to which we compared, including several novel candidates. Beyond driver gene ranking, feature analysis showed a remarkably selective enrichment of TSGs among large driver gene proteins, with the large TSGs functioning primarily in chromatin modification processes. Following this insight, CARNAF and other methods predict the presence of additional uncharacterized driver genes among the <1% of genes encoding very large proteins (top 5% in genome) that participate in chromatin biology.

## Results

**Figure 1:**
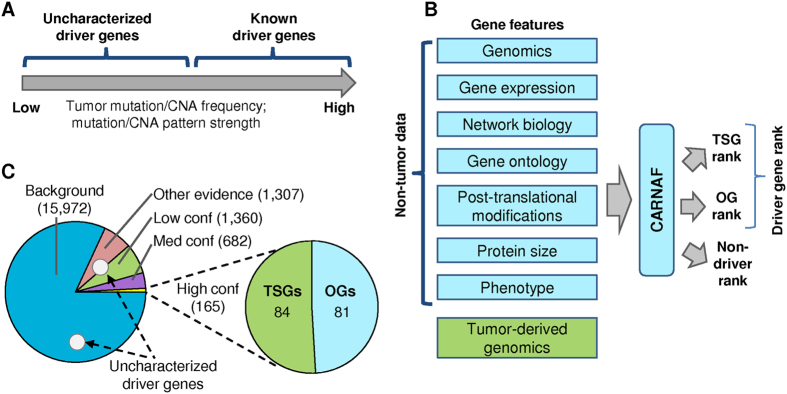
Approach for detection of infrequently mutated driver genes. (**A**) There is likely a long-tail of uncharacterized driver genes with infrequent somatic tumor aberrations or atypical mutation patterns. CNA–copy number alteration (deletions and gains). (**B**) Illustration of the CARNAF pipeline. A diverse set of gene-specific features are extracted and used for ranking genes as TSGs, OGs, or non-driver genes. (**C**) Breakdown of genes used for CARNAF training. 165 high confidence driver genes (84 TSGs and 81 OGs) are used as positive examples. Additional genes present in at least one of 15 pan-cancer/multi-tumor type studies used in this work are divided into medium confidence, low confidence, and other evidence drivers and are omitted from training (Online Methods). The remaining 15,972 background genes are used as negative examples for CARNAF training.

Many well-studied and known driver genes were originally identified by searching for higher than expected mutation rates. Thus, it is likely that the remaining uncharacterized driver genes exhibit infrequent or atypical mutation patterns (Fig. 1A, Supplementary Fig. 1). As driver genes are known to be enriched for specific properties^1^,^2^, methodical analysis of these traits can help focus the search on a smaller subset of candidate genes, and a machine learning approach that integrates both tumor data and other gene level traits may elucidate important driver gene traits.

### Gene features

CARNAF uses a broad set of gene properties. We extracted tumor-derived and non-tumor based gene features spanning genomic, transcriptomic, proteomic, functional, and phenotypic categories (Fig. 1B, Table 1, Supplementary Tables 1 and 2, and Online Methods). The tumor genomics features consisted of copy number variation data and four gene mutation patterns that are highly predictive of TSG and OG function^13^. A total of 131 features remained after removal of sparse and inter-correlated features (Online Methods).

### Driver and background gene datasets

Supervised machine learning methods require the construction of a labeled data set to train a classifier that can categorize new genes. For this, we defined three gene classes of interest: TSGs, OGs, and background genes (BGs) which are genes that are not known to act as drivers. As there is large variation among published lists of driver genes, we assembled 15 multi-tumor type driver gene sources to aid in label construction (Supplementary Tables 3 and 4). These studies included drivers present in at least one, and often more tumor types. From this set we selected 165 high confidence drivers with a known function that are present in at least one tumor type (84 TSGs, 81 OGs), 682 medium confidence drivers, 1,360 low confidence drivers, and 15,972 BGs (Fig. 1C and Online Methods).

### CARNAF methodology

CARNAF performs multi-class classification using a random forest, a robust predictive model composed of an ensemble of decision trees, each of which is trained on a subset of the training data^20^. The training set consists of 165 high confidence driver genes labeled as TSGs or OGs, and 15,972 BGs (Fig. 1c). Genes in the medium confidence, low confidence, and other evidence sets were excluded from training, since they may contain false positive results and often cannot be functionally categorized as TSGs or OGs. After training, every gene in the genome is assigned a posterior probability of being a TSG, OG or BG, such that the probabilities sum to one. The use of a large set of negative examples that is likely to include a small subset of mislabeled examples (genes that are labeled as BGs but have the potential to become driver genes) is known as positive-unlabeled (PU) learning in machine learning literature^21^.

Each gene was ranked according to the posterior probability of being a TSG, OG, or a BG as computed according to all trees that did not use the specific gene for training, out of 100,000 generated trees. Driver gene probabilities were obtained via the summation P(driver) = P(TSG) + P(OG). As the training data contains 97-fold more BGs than high confidence driver genes, we employed a stratified resampling with replacement approach where each tree used a training set with 165 high confidence driver genes and 165 BGs, as is commonly done in PU learning^22^. Additional details are provided in the Online Methods.

### Non-tumor genomics features improve detection of rare drivers

**Figure 2:**
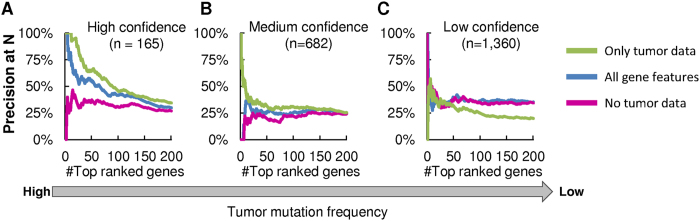
Use of non-tumor based features improves detection of rare driver genes. Precision at N shown for three gene sets: (**A**) high confidence driver genes, (**B**) medium confidence genes, and (**C**) low confidence genes. The 200 top ranked driver genes are shown sorted by rank. Going from left to right, the genes considered in each panel are excluded from subsequent panels. Precision in this scenario is equivalent to the fraction of detected genes. High confidence drivers, which are frequently mutated, are better detected using tumor genomics data. In contrast, non-tumor genomics data increases detection of candidate driver genes that are infrequently mutated.

As most computational methods identify driver genes by relying primarily on tumor data, we asked whether a large set of gene features beyond tumor data may be beneficial for detecting rare drivers. To this end, we compared CARNAF driver gene rankings using three different sets of features: all gene features, all gene features except tumor genomics, and only tumor genomics features (Fig. 2 and Supplementary Table 5). Prediction performance was evaluated as the ability to prioritize high, medium, and low confidence driver genes over BGs, with the medium and low sets expected to contain high false positive rates. As expected, precision at N shows that tumor genomics alone are ideal for detecting the highest confidence drivers, as these genes are frequently mutated (Fig. 2). In contrast, precision at N among the lower confidence sets corroborates that addition of non-tumor genomics features provides advantages when looking for infrequently mutated driver genes.

We further investigated several additional driver gene detection aspects. First, we verified that CARNAF results remained very similar when using slightly different training sets, indicating it is robust to the specific choice of training genes (Supplementary Table 6). Second, we verified that CARNAF accurately distinguished high confidence TSGs from OGs (Supplementary Fig. 2 and Supplementary Note). This was especially notable when using tumor genomics features (area under Receiver Operating Characteristic curve of 0.94 ± 0.02, out of bag estimation) since frequently mutated drivers have strong characteristic mutation patterns^1^. Third, the cumulative detection rate was superior when using non-tumor genomics features for all gene confidence sets (Supplementary Fig. 3). Fourth, we evaluated gene rankings in the absence of gene ontology features, as these can be biased towards well-studied genes. We observed a decrease in precision for low confidence genes (Supplementary Fig. 4 and Supplementary Table 5), suggesting that gene ontology features are useful for proposing rare drivers. Fifth, we demonstrated that although frequently mutated drivers are overrepresented among the high confidence drivers used for training (Fig. 1A), this has minimal impact on the top ranked genes (Supplementary Fig. 5, Supplementary Table 7, and Supplementary Note).

Finally, we performed manual literature curation of the top 15 ranked CARNAF driver genes (excluding the high confidence drivers) using all features and compared the results to 15 multi-tumor type cancer studies (Supplementary Tables 4 and 8). Strikingly, we found that the large majority of the 15 genes had substantial cancer-related evidence (Supplementary Table 8), often supported by genomics or functional assays. 4 (*SIRT1, TGFBR1, CDK1*, and *SMAD1*) of the 15 genes were not present in any of the multi-tumor type cancer studies. All of the latter 4 genes contain cancer-related evidence^23^,^24^,^25^,^26^,^27^,^28^,^29^,^30^,^31^, with *SIRT1* and *TGFBR1* also having documented genomic alterations^29^,^30^,^31^ (Supplementary Note).

### Contribution of individual features to driver gene detection

Random forests, which CARNAF uses for gene ranking, provide a measure of importance for each feature which quantifies its contribution to the classification process. The majority of top ranked features were confirmed to be those that are known to be cancer-related such as signal transduction, cell differentiation, cell proliferation, number of protein-protein interactions, predicted haploinsufficiency, specific phosphorylation events, and tumor mutation patterns (Supplementary Table 9). The total number of gene ontology terms also ranked high in feature importance, suggesting a potential bias towards high ranking of previously studied genes.

Comparison of feature distributions among TSGs, OGs, and BGs also identified known distinguishing features (Supplementary Tables 10 and 11), many of which were ranked as highly important for classification (Supplementary Table 9). Perhaps the most interesting finding is that TSGs encode significantly larger proteins than OGs (P = 1.27 × 10^−5^, Welch t-test). Among binary features, involvement in chromosome organization processes was a major differentiator between TSGs and OGs (*P* = 2.25 × 10^−6^).

### TSGs selectively encode very large driver gene proteins

**Figure 3:**
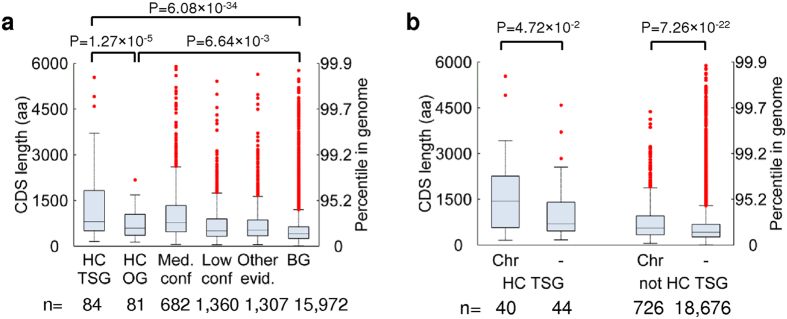
Large driver gene proteins are almost exclusively encoded by TSGs and primarily regulate chromosome organization. (**a**) Comparison of protein size distributions encoded by high confidence (HC) TSGs, high confidence OGs, medium confidence drivers, low confidence genes, other evidence genes that are present in at least one of 15 studies used in this work (Online Methods), and background genes (BGs). A high fraction of TSGs encode very large proteins. CDS–coding sequence. (**b**) Comparison of high confidence TSG and non-TSG protein size with respect to having a documented role in chromosome organization processes (Chr) based on gene ontology. Large TSG proteins are enriched for participation in chromosome organization processes. All P values are derived using the Welch t-test.

The observation that TSGs and OGs encode large proteins had been previously noticed but not thoroughly characterized^13^ (Fig. 3a). Upon deeper investigation we detected a profound enrichment of TSGs and depletion of OGs, specifically among the largest proteins in the high confidence set (*P* = 8.56 × 10^−6^, hypergeometric test using the 30 largest driver proteins) (Table 2). This TSG enrichment among large driver proteins is particularly fascinating as it has been considered an artifact to some extent by previous studies^10^.

To alleviate the concerns that this observation is an artifact of previously suggested confounding factors, we evaluated several hypotheses as to why TSGs encode very large proteins, specifically in comparison to OGs, and found no association between any of the factors and protein size. Assessed explanations included expression levels, DNA replication timing, protein connectivity, gene deletion frequency, gene mode of inactivation, gene essentiality, gene duplications, and presence in specific gene ontology terms or pathways (Supplementary Figs 6–13, Supplementary Tables 12 and 13, and Supplementary Note).

### Large TSGs are frequently involved in chromatin modification

Noting that chromosome organization was the strongest feature associated with TSGs compared with OGs (Supplementary Table 10), we hypothesized that TSG protein size and chromosome organization are related. Indeed, involvement in chromosome organization was the feature most strongly associated with TSG size, with chromosome organization TSGs having a 3.8 fold-larger median coding sequence length than non-chromosome organization TSGs (P = 0.047, Welch t-test) (Fig. 3b, and Supplementary Figs 14 and 15).

**Figure 4:**
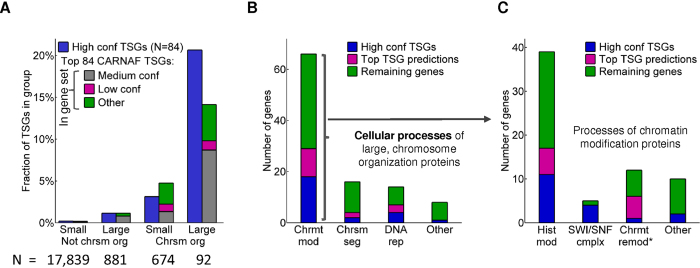
CARNAF and other methods predict an enrichment of uncharacterized TSGs among very large chromatin regulators. (**A**) The abundance of high confidence TSGs and CARNAF predicted TSGs encoding very large (top 5% in genome) and small proteins (the remaining 95%) with respect to participation in chromosome organization processes. The top 84 CARNAF TSG predictions, using all features and excluding the high confidence driver gene set, were selected to match the abundance of TSGs in the high confidence set (n = 84). CARNAF predictions that overlap with the medium and low confidence driver gene sets are shown. Chr – chromosome organization biological process, according to gene ontology. (**B**) Prominent cellular processes for the 92 large, chromosome organization proteins. The fraction of high confidence and CARNAF predicted TSGs in each category is displayed. Categories are not mutually exclusive. (**C**) Specific cellular processes of the 66 genes annotated as involved in chromatin modification. Categories are mutually exclusive. Abbreviations: Chrsm–chromosome; Chrmt – chromatin; Chrmt mod – chromatin modification; Chrsm seg – chromosome segregation; DNA rep – DNA repair; Hist mod – histone modification; SWI/SNF cmplx – SWI/SNF complex; Chrmt remod* – other chromatin modification not annotated as histone modification or SWI/SNF complex.

The above suggests that the simple intersection of large protein size with a role in chromosome organization may pinpoint a small set of genes with potentially uncharacterized driver gene function. In fact, 19 out of the 84 high confidence TSGs (23%) are among the 92 genes that encode the top 5% largest proteins in the genome and are involved in chromosome organization (Fig. 4A). This is a 62-fold enrichment compared to TSG prevalence among the remaining genes (*P* = 2.3 × 10^−27^, hypergeometric test). In contrast, only a single high confidence OG is found among these 92 genes.

CARNAF as well as other pan-cancer/multi-tumor type studies predict an additional high concentration of putative driver genes among this focused gene set, primarily of TSG function (Fig. 4A, Supplementary Figs 16 and 17, Supplementary Tables 4 and 5, and Supplementary Note). Similar to the 84 high confidence TSGs, 13 out of the top 84 CARNAF TSG predictions encode large chromosome organization proteins, 8 of which are present in the medium confidence set (*CHD8, KAT6A, KMT2A, KMT2B, KMT2E, NIPBL, NSD1*, and *TAF1*). Two additional genes out of the 13 (*INO80* and *RERE*) were detected as TSGs by TUSON^13^ (Supplementary Table 4), and the remaining 3 (*PRKDC, PSME4*, and *SUPT6H*) had little or no evidence among the 15 studies used in this work. *PRKDC* was only detected by the author implementation of a simple TSGs versus OGs mutation-based classifier^1^, *PSME4* was not present in any source, and *SUPT6H* was ranked far below the significance threshold in TUSON (rank 709).

A review of literature for *SUPT6H* and *PRKDC* provides a degree of support for a potential driver gene role. *SUPT6H* encodes a histone chaperone that acts as a transcription elongation rate enhancer. The gene may suppress breast cancer as its protein levels are inversely correlated with breast cancer malignancy. It also promotes estrogen receptor-dependent transcription and chromatin structure maintenance^32^. *PRKDC* encodes a serine/threonine-kinase involved in DNA repair and recombination, with little current documentation for driver mutations within the gene. However, PRKDC inhibition sensitizes cells to irradiation^33^ and is synthetic lethal in *MYC* dependent cancers^34^ and with the mismatch repair gene *MSH3*^35^. The latter studies suggest non-oncogene addiction to *PRKDC*.

The observation that a high percentage of very large chromosome organization proteins are driver genes, specifically TSGs, is consistent with the vastly growing appreciation of mutated epigenetic regulators as causal cancer drivers^36^,^37^. Indeed, of the 92 genes in the above category, the majority (n = 66; 72%) are involved in chromatin modification according to gene ontology^38^ (Fig. 4B), with many (n = 39, 42%) specifically linked to various types of histone protein modification, primarily methylation and acetylation (Fig. 4C and Online Methods). Consistent with the above, the majority of high confidence TSGs (17 of 19) and top predicted TSGs (11 of 13) among very large chromosome organization proteins (top 5% in genome) are annotated as involved in chromatin modification. Among the 13 CARNAF predicted genes, there are 4 histone methyltransferases (*KMT2A, KMT2B, KMT2E*, and *NSD1*), 2 histone acetyltransferases (*KAT6A* and *TAF1*), and 5 genes involved in chromatin remodeling (*CHD8, PSME4, RERE, SUPT6H, and INO80* which is also involved in DNA repair and chromosome segregation)^38^. The remaining 2 genes are involved in DNA repair (*PRKDC*) and chromosome segregation by loading the cohesion complex onto chromatin (*NIPBL*)^38^.

## Discussion

In this study we used diverse gene properties beyond tumor genomes to detect cancer driver genes and classify their mode of action. This integrative approach enabled us to detect literature-supported driver genes that are not present among a large compendium of driver genes derived from over a dozen efforts. We show that the largest driver genes are almost exclusively TSGs, with a remarkably dense concentration of known and putative drivers among very large proteins involved in chromatin modification. These findings highlight a small subset of candidate genes for focused experimental investigation, specifically as driver genes that modify the cancer epigenome.

The use of non-tumor genomics gene-level knowledge has been previously shown to aid in driver gene detection; however, this was typically done using a small set of select features. For example, MutSigCV uses DNA replication timing and cell line expression levels^10^, ActiveDriver uses phosphorylation site knowledge^39^, and HotNet2 uses protein network knowledge^15^. One exception, MAXDRIVER, uses a larger set of features, although only to detect copy number-based drivers in select tissues^19^. By integrating diverse gene knowledge in a pan-cancer framework, CARNAF found additional putative driver genes beyond what was previously found by other computational efforts.

Our work argues for a deeper mechanistic investigation of the link between protein size and cancer driving potential. It appears that while driver genes have high protein-protein connectivity, protein network centrality is not associated with TSG protein size and does not explain why TSGs encode large proteins compared to OGs. Perhaps evolutionary considerations and alternative gene function hypotheses may offer insight. Functionally, large genes and proteins tend to be evolutionarily conserved^40^, exhibit increased essentiality^41^, and have less redundancy and gene duplications^42^ as there is selective pressure for proteins to be short in order to preserve resources^43^. The above may guide further exploration.

The focus on very large proteins involved in chromatin modification as an enriched pool of candidate TSGs targets roughly 0.3% of the protein-coding genes in the genome for potential validation. This emphasis is consistent with the recently increased appreciation of mutated epigenetic regulators as cancer drivers^36^,^37^. The above gene pool, beyond containing characterized driver genes, contains many additional genes predicted as drivers by CARNAF and other methods. Thus, this suggests that our knowledge of cancer-causing mutations to epigenome modifiers is potentially far from complete.

The TSG predictions in this highly enriched set of genes, even if functionally incorrect, may still be important cancer driver genes. For example, CARNAF predicts that the very large (top 3% in genome) histone acetyltransferase encoded by *KAT6A* functions as a TSG (ranked 10^th^ top TSG prediction, Supplementary Table 8). While it was hypothesized that KAT6A may suppress cancer in response to severe DNA damage^44^, stronger evidence suggests an OG role as it is very frequently amplified and has been experimentally shown to act as a breast cancer oncogene and senescence inhibitor^44^,^45^,^46^,^47^.

Machine learning methods such as CARNAF provide a major advantage by systematically integrating many features for gene ranking. Beyond this study, additional scenarios can be envisioned, for example by using alternative training sets (as done previously^13^), performing lineage specific rather than pan-cancer analysis (as done previously^48^), limiting use of features with missing data or bias (e.g. gene ontology annotations towards well studied genes), including additional gene features, using a different learning methodology, and modifying various other parameters.

In this study we used machine learning techniques on a large set of publically available data to highlight a targeted set of genes for further validation as cancer drivers. Ultimately, our work suggests the presence of numerous uncharacterized, epigenetics-based driver genes, most of which are predicted TSGs, among very large regulators of chromatin structure.

## Methods

### Data Preparation

#### Selection of driver genes to be used as training examples

We constructed an integrated list of high confidence, protein-coding driver genes covering both mutation-based and copy number alteration driver genes. The data sources used to compile this list and the background genes list consisted of over a dozen multi-tumor type studies and databases (Supplementary Tables 3 and 4). These sources included drivers present in at least one, and often more, tumor types. Considerations for inclusion comprised source reliability, presence in multiple sources, and source confidence in the given gene. The list does not consider driver genes resulting from chromosomal rearrangements. The high confidence set was built in several sequential steps as described below.

First, we included all of the mutation driver genes (71 TSGs, 54 OGs) and copy number alteration driver genes (3 TSGs, 10 OGs) reported in Vogelstein *et al*. as these represent well-known, manually curated driver genes^1^.

Second, we supplemented the TSGs that result from deletions. We added the two genes (*FANCD2* and *TSC2*) that were labeled by the Cosmic Cancer Gene Census (CGC)^49^ and by at least two of the following three sources: TAG DB^3^, TSGene^4^, and our 20/20 classifier implementation, a mutation-pattern based classifier that labels genes as TSGs or OGs^1^ (briefly described in Supplementary Table 3). Next, we added the four remaining genes (*CDKN1B, FAT1, IKZF2, MYCN,* and *PARK*) that were curated by Zack *et al*.^45^ as known frequently deleted TSGs.

Third, we supplemented the OGs that result from amplification. We added the 12 remaining genes (*BCL2L1, CCNE1, CDK4, CDK6, E2F3, IGF1R, MCL1, NEDD9, PAX8, SOX2, TERT*, and *ZNF217*) curated by Zack *et al*. as known frequently amplified OGs. We then added the four remaining genes (*AKT2, JUN, MITF,* and *REL*) that were present in both in the CGC and Santarius *et al*.^50^.

Fourth, we supplemented the mutation-based drivers by curating the remaining genes that were identified by the highest amount of mutation-based methods. All genes present in six or seven mutation methods were already included. Of the four remaining genes present in five out of seven mutation methods, we assigned two (*CDK12* and *CTCF*) as TSGs with sufficient confidence based on manual literature curation. Of the 15 remaining genes detected by four mutation-based methods, we assigned two (*ELF3* and *ZFHX3*) as TSGs and two (*RAC1* and *TBX3*) as OGs based on manual literature curation.

Finally, we removed *MYCN* from the list as it may serve as both a TSG and OG according to the literature. Thus, *MYCN* was not used as a driver gene in the high confidence set.

The resulting high confidence set consisted of 84 TSGs and 81 OGs for a total of 165 driver genes, which were present in at least one tumor type. TSG mode of inactivation consisted of 46 mutation-based TSGs, 7 deletion-based TSGs, and 31 TSGs spanning either form of inactivation. OG mode of activation consisted of 46 mutation-activated OGs, 23 amplification-activated OGs, and 12 OGs that can be activated by either method.

#### Selection of background genes

We selected 19,486 protein-coding genes to be used in the study. The genes were derived by intersecting protein-coding genes from dbNSFP v2.4, the Gene Ontology Consortium^51^ (downloaded from http://geneontology.org on January 29^th^, 2015), and genes from Uniprot^52^ (http://www.uniprot.org/, downloaded on January 4, 2015) for which we could retrieve coding sequence lengths to ensure known proteins. The full list of genes can be found in Supplementary Table 1.

CARNAF requires a set of genes with little or ideally no cancer evidence to be used as negative examples. To this end, we removed the 165 high confidence driver genes and 3,349 additional genes that were reported in at least one source (Supplementary Table 4a) from the above. This resulted in 15,972 background genes (BGs) to be used as negative examples for classifier training.

#### Selection of medium and low confidence driver gene sets

We created medium and low confidence driver gene sets to evaluate CARNAF driver gene detection beyond high confidence drivers, the latter being the training set (n = 165). Both sets consist of non-consensus genes and are expected to contain high rates of false positives.

The medium confidence set (n = 682) includes all genes present in at least one of the following nine genomics-based sources: Zack *et al*. (deletion/amplification)^45^, CGC (deletion/amplification/mutation)^49^, Santarius *et al*. (amplification)^50^, Lawrence *et al*. (mutation)^9^, HotNet2 (mutation)^15^, MuSiC (mutation)^53^, OncoDriveClust (mutation)^54^, OncoDriveFM (mutation)^55^, and ActiveDriver (mutation)^39^. TUSON^13^ was not used in the above gene sets as it was the origin of our genomics features. Genes from the high confidence set were excluded.

The low confidence gene set (n = 1,360) includes genes present in at least one of the following sources and not in the medium and high confidence sets: TagDB^3^, TSGene^4^, our own 20/20 rule implementation^1^, and genes ranked high (absolute effect >3 in Supplementary Table 4b) as biomarkers in a high-throughput cell line study^5^.

#### Feature extraction-data sources

dbNSFP v3.0b2a was used to retrieve protein-protein interaction (PPI) data^56^,^57^. PPI data included IntAct (downloaded on March 27, 2015) and BioGRID (version 3.3.122). dbNSFP v2.4 was used to retrieve GO slim terms. The GO slim terms had a Gene Ontology Consortium validation date of September 27, 2013. Full GO terms were not used as they are relatively sparse features. dbNSFP v2.4 was also used to retrieve an estimated probability of haploinsufficiency per gene^58^ and gene essentiality predictions based on homology with the Mouse Genome Informatics database^41^. Essentiality predictions had three categories: essential, non-essential but phenotype-changing, and all other genes. The coding sequence was determined as the longest isoform within the gene, as retrieved from UniProt on January 4, 2015. Ensembl BioMart was used on November 26, 2014 to retrieve the GC percent per gene and the number of transcripts per gene. Chromatin compartment and DNA replication time were retrieved from Lawrence *et al*.^10^. Duplicate gene data was retrieved from Ouedraogo *et al*.^42^. Healthy tissue gene expression data was derived by Fagerberg *et al*.^59^ and retrieved from EMBL-EBI ArrayExpress (http://www.ebi.ac.uk/arrayexpress/experiments/E-MTAB-1733/). Post-translational modification (PTM) data was downloaded from PhosphoSitePlus^®^, www.phosphosite.org, on September 24, 2014^60^. All acetylation, SUMOylation, trimethylation, ubiquitination PTMs were chosen on lysine residues since such PTMs rarely occurred on other residues, thus using non-lysine residues would result in sparse features. Likewise, all tyrosine modifications were of phosphorylation type.

#### Tumor-derived genomics features

Four tumor mutation-pattern features were used in the study: (a) the entropy score (a measure of the randomness of mutation distribution across a gene), (b) the ratio of loss-of-function mutations to benign mutations per gene, (c) the ratio of splice site mutations to benign mutation per gene, and (d) the ratio of missense mutations predicted to have high functional impact by PolyPhen2 Hum-Var^61^ to benign mutations per gene. These four features were shown to be highly informative for TSG and OG classification, and were extracted from the original study^13^.

Somatic gene amplification and deletion frequency, specifically GISTIC2 p-values^62^, was extracted from the above study^13^.

#### Feature extraction-computations

Several of the features we used required some computation, as detailed below.

Genomic density: The genomic density of a gene was determined as in previous work by quantifying the number of genes that reside within 4Mb upstream or downstream from the center chromosomal position of the gene, and then dividing by the mean of this number^63^.

Number of PPIs: The number of PPIs per gene was calculated as the average number of interactions present in both sources (BioGrid and IntAct) as retrieved from dbNSFP v3.0b2a.

Betweenness centrality: Betweenness centrality was calculated by running the Brandes algorithm^64^, using the iGraph R package^65^, on the BioGRID PPIs (Release: 3.2.111), which were accessed via the rTRM package^66^ for this purpose.

#### Non-tumor genomics features considered but not used

An effort was made to reduce feature redundancy by avoiding the use of multiple data sources for the same feature type. For example, we did not use pathway data from the Kyoto Encyclopedia of Genes and Genomes to prevent redundancy with GO slim biological process terms.

In addition, we evaluated protein and mRNA stability data but ultimately did not include these features as the data was typically incomplete. Regarding mRNA turnover rates, human datasets have half-life data for only up to approximately 50% of protein-coding genes^67^,^68^,^69^. Likewise, protein stability data exists for roughly 40% of genes^70^,^71^. In contrast to humans, global mRNA stability data does exists for the majority of mouse genes in ES cells^72^.

We also evaluated InterPro protein features, retrieved from Ensembl BioMart on November 26, 2014. InterPro contains data on protein families, domains, and functional sites. However, as only 1 of the 7,132 extracted features passed feature selection due to high sparsity, we opted to omit InterPro features.

#### Total features used and feature removal

A total of 131 features were used by CARNAF after two sequential feature removal steps: sparse feature removal and correlated feature removal.

#### i) Sparse features

Identification and removal of sparse features was performed using the R caret package^73^. Sparse features were defined as features satisfying two criteria: the ratio between the frequency of the first and second most common values was larger than 97/3, and the percentage of unique values out of the number of genes was smaller than 3%. A value of ‘unknown’ was also considered a unique value, thus leading to the omission of features with substantial levels of missing values.

#### ii) Removal of correlated features

To avoid redundancy, features were omitted if they were highly correlated to other features using the R caret package. Two features were considered highly correlated if they had a Spearman correlation coefficient with an absolute value >0.95. For each pair of correlated features, the feature with the greater mean absolute correlation with the remaining features was removed. After this stage, 131 features remained for use by CARNAF.

#### Missing data imputation

Missing values for features that were not omitted in the previous stage were imputed via the k-nearest neighbor procedure using the R caret package. For each gene, missing values were imputed according to the mean value of its 5 nearest neighbors, where similarity was measured using Euclidean distance. Features that could not be imputed because the same feature was missing in all 5 nearest neighbors were imputed as the median using all genes in the sample.

#### Additional data sources used in study, but not in CARNAF

Tissue expression data in binary format was used in Supplementary Table 12c-d. The expression data source GNF/Atlas (BioGPS) was downloaded from Ensembl BioMart on October 1, 2013 and retrieved via dbNSFP v3.0b2a. Cancer cell line expression data was retrieved from Lawrence *et al*.^10^. The data is presented in Supplementary Table 2.

### Carnaf Methodology

#### PU learning

CARNAF employs PU learning^20^,^75^,^76^, wherein genes without cancer evidence are treated as negative labels in an ensemble learning approach, where many classifiers are trained on different subsets of the data. This approach has been compared favorably with methods that do not make use of unlabeled or negative example genes, such as Endeavour^17^, Toppgene^77^, or density estimators^78^. The theoretical merits of this approach have been previously discussed^22^.

#### Classification

Classification of genes as TSGs, OGs, or non-driver genes was performed using a random forest classifier^21^. Random forests enable multi-class classification and have been demonstrated to often outperform other well-known classifiers in a variety of predictive modeling domains^79^. Briefly, a random forest is an ensemble of a large number of decision tree classifiers, each of which is assigned to a random bootstrapped sample of the data as its training set. Random forest classifiers also compute feature importance, in addition to classification, by quantifying the mean decrease in node impurity (also known as *Gini index*) gained by splitting a node in a decision tree according to each feature. We employed the R randomForest package^80^ using 100,000 trees for each classification task and the caret R package for parameter tuning^73^. For each gene, the posterior probability of being a TSG, OG, or background gene was computed according to all the classifiers to which it was not assigned. This procedure is known as out of bag estimation.

#### Unbalanced classes

The training data contains 97-fold more background genes than driver genes. We used a stratified down-sampling approach to deal with this class imbalance, where each decision tree in the ensemble was assigned a random subset of the data. Data subsets consisted of the 165 high confidence driver genes and 165 randomly selected negative example genes, where each was sampled with replacement using the “sampsize” option of the R package randomForest. A similar approach was previously recommended in a PU learning setting^22^. We note that while the estimated posterior driver gene probabilities are useful for gene ranking, they should not be regarded as the true posterior probability of being a driver due to the down-sampling procedure.

#### Receiver operating characteristic (ROC) curve for TSG vs OG classification

ROC curves were calculated using the 84 TSG and 81 OG driver genes. TSG labels were set to 1 and OG labels to 0. The probability of the label equaling one was defined as


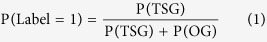


## Additional Information

**How to cite this article**: Waks, Z. *et al*. Driver gene classification reveals a substantial overrepresentation of tumor suppressors among very large chromatin-regulating proteins. *Sci. Rep.* **6**, 38988; doi: 10.1038/srep38988 (2016).

**Publisher's note:** Springer Nature remains neutral with regard to jurisdictional claims in published maps and institutional affiliations.

## Supplementary Material

Supplementary Information

Supplementary Dataset 1

Supplementary Dataset 2

Supplementary Dataset 4

Supplementary Dataset 5

Supplementary Dataset 8

Supplementary Dataset 12

Supplementary Dataset 13

## Figures and Tables

**Table 1:** Gene-specific features used in study.

Feature category (# features)	Description
*Genomic*	
GC percent (1, 1)	GC content of gene, including introns
Genomic density (1, 1)	Number of genes that are present ≤4 Mb from gene center
DNA replication time (1, 1)	Stage in cell cycle in which gene is replicated
Number of transcripts (1, 1)	Number of transcripts per gene
Chromatin compartment (1, 1)	Extent that the chromatin compartment of the gene is open or closed (HiC experiment)
*Gene expression - healthy tissue*	
Tissue RNA levels (27, 27)	Expression levels from 27 different tissues
Median across tissues (1, 1)	Median expression level across tissues
Variation across tissues (1, 1)	Coefficient of variation (mean divided by standard deviation) across the 27 tissues
*Protein size*	
Coding sequence length (1, 1)	Number of amino acids in longest gene isoform
*Post-translational modification*	
Number of modified residues (14, 10)	Number of acetylation, methylation (mono, di, & tri), phosphorylation, SUMOylation, and ubiquitination sites (normalized by CDS length)
*Network biology*	
Number of PPIs (1, 1)	Number of protein-protein interactions
Gene duplication (1, 1)	Is the gene a duplicate gene
Betweenness centrality (1, 1)	Measure for centrality within networks as quantified by frequency in shortest paths between nodes (proteins).
*Gene ontology*	
GO slims biological process (70, 36)	Biological process in which the gene is involved. Gene Ontology (GO) slims are high level gene ontology terms.
GO slims molecular function (34, 16)	Specific function of encoded proteins
GO slims cellular component (42, 19)	Spatial location of encoded proteins
Number of total GO slim terms (4, 4)	Number of total GO slims terms and total per each GO category
*Phenotype*	
Predicted haploinsufficiency (1, 1)	Estimated probability of haploinsufficiency of the gene
Predicted essentiality (1, 1)	Essential gene or non-essential but phenotype-changing based on mouse homology
*Tumor-derived genomics*	
Mutation patterns (4, 4)	Four features: Mutation clustering estimation (distribution entropy) and ratio of predicted loss-of-function, damaging missense, and splice site mutations to benign mutations
Copy number alteration (2, 2)	Somatic gene amplification and deletion frequency

A diverse set of feature classes were used in the study. The number of features within each category before and after feature selection is presented in parentheses (before, after). 131 features remained after feature selection (Online Methods and Supplementary Tables 1 and 2).

**Table 2:** Large driver proteins are encoded almost exclusively by TSGs.

Symbol	Type	CDS length (aa)	Percentile in genome
KMT2D	TSG	5537	99.9%
KMT2C	TSG	4911	99.8%
FAT1	TSG	4588	99.7%
CSMD1	TSG	3565	99.5%
BRCA2	TSG	3418	99.4%
ATM	TSG	3056	99.2%
APC	TSG	2843	99.1%
NF1	TSG	2839	99.1%
SETD2	TSG	2564	98.8%
NOTCH1	TSG	2555	98.8%
CIC	TSG	2514	98.7%
ATRX	TSG	2492	98.7%
NOTCH2	TSG	2471	98.6%
CREBBP	TSG	2442	98.6%
NCOR1	TSG	2440	98.6%
EP300	TSG	2414	98.5%
ARID1A	TSG	2285	98.3%
ARID1B	TSG	2236	98.2%
**MED12**	**OG**	**2177**	**98.1%**
TET2	TSG	2023	97.7%
BRCA1	TSG	1884	97.2%
ARID2	TSG	1835	97.0%
TSC2	TSG	1807	96.9%
BCOR	TSG	1755	96.6%
PBRM1	TSG	1689	96.2%
SMARCA4	TSG	1681	96.2%
**DNMT1**	**OG**	**1678**	**96.2%**
**ALK**	**OG**	**1620**	**95.9%**
**SETBP1**	**OG**	**1596**	**95.8%**
KDM5C	TSG	1560	95.6%

List of the high confidence driver genes encoding the 30 largest proteins. CDS – coding sequence length.
