# Supplementary material for: Driver gene classification reveals a substantial overrepresentation of tumor suppressors among very large chromatin-regulating proteins
Source: Sci Rep. 2016 Dec 23;6:38988. doi: 10.1038/srep38988 (PMC5180091; doi:10.1038/srep38988)
Supplement: Supplementary Information [file srep38988-s1.pdf]

Supplementary Information for

Driver gene classification reveals a substantial overrepresentation of tumor suppressors among very large chromatin-regulating proteins

Zeev Waks[1*], Omer Weissbrod[1*], Boaz Carmeli[1], Raquel Norel[2], Filippo Utro[2], Yaara Goldschmidt[1]

[1]Machine Learning for Healthcare and Life Sciences, IBM Research – Haifa, Mount Carmel Campus, Israel
[2]Computational Biology Center, IBM T. J. Watson Research, Yorktown Heights, NY 10598, USA
*Corresponding authors: zeevw@il.ibm.com, omerw@il.ibm.com

Supplementary Note

1) Functional driver gene classification

CARNAF was also able to distinguish TSGs from OGs without using tumor features at relatively high accuracy (area under curve (AUC) = 0.79± 0.04, std. computed using 10^6 bootstrap iterations) (Supplementary Fig. 2). Comparatively, classification using only tumor genomics features (AUC = 0.94 ± 0.02) was significantly better (P = 4.46×10^{-4}, computed by a permutation test with 10^6 iterations). The combined classifier was expectedly in-between the latter two (AUC = 0.90 ± 0.02). Similar results were seen when using the less biased feature set which omits gene ontology features (Supplementary Fig. 4).

The strong performance using mutation features is not surprising as evaluation was performed using high confidence drivers, most of which are frequently mutated. Such TSGs harbor frequent loss of function mutations and deletions, in contrast to mutation clustering and gene amplifications for OGs. However, this performance cannot be generalized to infrequently mutated drivers due to weaker mutation patterns.

2) Curation of top ranked genes – TSG/OG classification

In contrast to driver gene detection recall rates, functional classification by CARNAF and the 15 data sources used in the study was somewhat less accurate compared to the literature. For CARNAF, functional predictions for 5 out of the 12 genes that had supporting functional evidence, TSG or OG, in the literature were contradictory. Using functional annotations from the 15 multi-tumor type sources used in the study, only 2 out of the 8 genes had functional predictions that were discordant. These results illustrate a degree of accuracy, but have a lower performance than the TSG versus OG classification among the high confidence driver genes (Supplementary Fig. 2), although this is not surprising as infrequently mutated drivers generally display weaker mutation patterns and cancer-like features, and additionally the sample size is small. In addition, various driver genes can function as both TSGs and OGs[1–3], a factor that also can lower true classification accuracy.

3) Curation of 4 genes ranked high by CARNAF that eluded the reference studies used in this work

We curated the top 15 CARNAF driver genes using all features (excluding the high confidence drivers) and found 4 genes (*SIRT1*, *TGFBR1*, *CDK1*, and *SMAD1*) with cancer-related evidence that were not present in the 15 multi-tumor type studies to which we compared (Supplementary Tables 4 and 8).

Of these 4 genes, *SIRT1* and *TGFBR1* also have supporting genomic alterations[4,5]. *SIRT1* encodes an NAD-dependent histone deacetylase that links intracellular energetics with transcriptional regulation and may both suppress and promote tumors depending on the context[6], with loss-of-function mutations and allelic loss being characterized in tumor cell lines[4]. TFGBR1 is a serine/threonine kinase receptor that mediates TGF-β signaling, with a meta-analysis of 35 studies proposing that two specific mutations are associated with cancer susceptibility[5].

The other two genes may be infrequently mutated drivers. *CDK1* encodes a serine/threonine kinase that controls cell cycle progression. Although little direct mutation evidence exists, *CDK1* has been associated with chemotherapy resistance[7,8], with high activity being correlated with poor colorectal cancer prognosis[9,10]. Finally, *SMAD1* mediates bone morphogenetic protein signaling and is involved in various cancer-relevant processes. Phosphorylated, hyperactive SMAD1 may promote epithelial–mesenchymal transition in lung cancer cell lines[11] and elevated phosphorylation has been observed in breast cancer metastases[12].

4) Evaluation of potential origins of large TSG protein size in relation to OGs

We evaluated several hypotheses why TSGs frequently encode very large proteins, particularly in comparison to OGs, to ensure it is not a known artifact of previously described findings.

First, as large genes are prone to high mutation rates due to low expression and late DNA replication, we verified that there is little association between TSG protein size and replication time ($R = 0.06$, $P = 0.58$; log scale) or expression ($R = 0.04$, $P = 0.70$; log scale)[13] (Supplementary Fig. 6).

Second, it has been proposed that driver genes encode large proteins given their high connectivity in protein networks[14], as highly connected proteins tend to be large (Supplementary Fig. 7). However, TSG protein size was not significantly correlated with either the number of protein-protein interactions or with presence in shortest network paths after accounting for testing of more than one hypothesis (Supplementary Fig. 8). Moreover, connectivity values were similar between TSGs and OGs, despite the former encoding a distribution of larger proteins (Supplementary Fig. 9).

Third, there is often reduced selective pressure against deletions of large genes which can lead them to be mistaken as TSGs. This is because large genes, which often encode large proteins, tend to reside in regions of low gene density[15]. However, we did not find significant association between TSG protein size and deletion frequency (Supplementary Fig. 10), and there were also no differences between TSG size among different modes of inactivation (Supplementary Fig. 11).

Fourth, essential genes and non-duplicate genes are known to encode large proteins[16,17]. However, differences between TSGs and OGs were minor and the abundance of duplicate genes was small in both driver gene classes (Supplementary Figs. 12 and 13).

Finally, we examined gene ontology categories in which TSGs reside and found that no biological process, function, localization or expression in particular tissues could explain TSG size (Supplementary Tables 12 and 13). Thus, the tendency of TSGs to encode very large proteins is not well explained by the hypotheses above.

5) Assessment of gene enrichment among very large, chromosome organization genes – HotNet2 evaluation

Similar to CARNAF, HotNet2 is a computational-based method aimed at detecting rare driver genes. Thus, we specifically analyzed the significantly mutated genes found by HotNet2[18], a method aimed at detecting rare drivers, to evaluate if they also contain an enrichment of large, chromosome organization proteins.

Of the 104 HotNet2 genes that were not in our high confidence driver gene set, 22 (21.2%) were among the top 5% largest proteins in the genome, of which 8 (7.7%) were also chromosome organization proteins, an 18-fold enrichment ($P = 3.22\times10^{-8}$, hypergeometric test) compared to the remaining genome-wide concentration of HotNet2 genes. This further supports the presence of uncharacterized driver genes in this focused gene pool. Perhaps not surprisingly given the different methodologies, there was little overlap between the top ranked genes of CARNAF and HotNet2 (Supplementary Fig. 17). Similarly, the majority (54%) of the 104 HotNet2 genes were also not present among the 3458 genes in the remaining 14 data sources used in the study to build the high confidence drivers, medium confidence driver genes, and background gene sets (Supplementary Table 4a).

6) Effect of down-sampling on gene rankings

The estimated posterior probability of being a TSG, OG, or BG is computed by an ensemble of classifiers. The training set for each classifier was down-sampled such that it contains the same number of driver (n=165) and background genes (n=165). This sampling does not preserve the ratio of driver genes to background genes. Below, we show that this procedure does not alter the ranking of driver genes. For ease of presentation, we consider a single classifier, with the generalization to an ensemble being relatively straightforward.

Denote $\mathrm{Y_i}$ as the (estimated) driver gene label of gene i (as estimated by a classifier when excluding this gene from the training set), where $\mathrm{Y_i} = 1$ if gene i is a driver gene, and $\mathrm{Y_i} = 0$ otherwise, denote $\mathrm{X_i}$ as the features vector of gene i, and denote $\mathrm{P(Y_i = 1 \mid X_i = x_i)}$ as the posterior probability of gene i being a driver gene. For each gene j, we introduce a binary selection variable $\mathrm{S_j}$, such that $\mathrm{S_j} = 1$ if gene j was included in the training set of the classifier. Since the classifier training set consists only of the subset of selected genes, the actual quantity estimated by the classifier when performing prediction for gene i is $\mathrm{P\left(Y_j = 1 \mid S_j = 1, X_j = x_j\right)}$.

Below we show that $\mathrm{P}(\mathrm{Y_i} = 1 \mid \mathrm{X_i} = \mathrm{x_i}) > \mathrm{P}(\mathrm{Y_j} = 1 \mid \mathrm{X_j} = \mathrm{x_j})$ whenever $\mathrm{P}(\mathrm{Y_i} = 1 \mid \mathrm{S_i} = 1, \mathrm{X_i} = \mathrm{x_i}) > \mathrm{P}(\mathrm{Y_j} = 1 \mid \mathrm{S_j} = 1, \mathrm{X_j} = \mathrm{x_j})$. This indicates that driver genes ranking remains the same regardless of down-sampling.

The central premise for the above is that S_i is conditionally independent of X_i given Y_i, which stems from the fact that the down-sampling procedure uses only the observed labels to decide if a gene is to be included in the training set. By invoking Bayes rule and the conditional independence assumption, we obtain

$$\mathrm{P}(\mathrm{Y_i} = 1 \mid \mathrm{S_i} = 1, \mathrm{X_i} = \mathrm{x_i}) = \frac{\mathrm{P}(\mathrm{Y_i} = 1 \mid \mathrm{X_i} = \mathrm{x_i})\mathrm{P}(\mathrm{S_i} = 1 \mid \mathrm{Y_i} = 1)}{\sum_{\mathrm{y'}} \mathrm{P}(\mathrm{Y_i} = \mathrm{y'} \mid \mathrm{X_i} = \mathrm{x_i})\,\mathrm{P}(\mathrm{S_i} = 1 \mid \mathrm{Y_i} = \mathrm{y'})}. \quad (1)$$

Assuming without loss of generality that gene i is ranked higher than gene j, we have $\mathrm{P}(\mathrm{Y_i} = 1 \mid \mathrm{S_i} = 1, \mathrm{X_i} = \mathrm{x_i}) > \mathrm{P}(\mathrm{Y_j} = 1 \mid \mathrm{S_j} = 1, \mathrm{X_j} = \mathrm{x_j})$. By using Equation 1, we obtain

$$\frac{\mathrm{P}(\mathrm{Y_i} = 1 \mid \mathrm{X_i} = \mathrm{x_i})\mathrm{P}(\mathrm{S_i} = 1 \mid \mathrm{Y_i} = 1)}{\sum_{\mathrm{y'}} \mathrm{P}(\mathrm{Y_i} = \mathrm{y'} \mid \mathrm{X_i} = \mathrm{x_i})\,\mathrm{P}(\mathrm{S_i} = 1 \mid \mathrm{Y_i} = \mathrm{y'})} > \frac{\mathrm{P}(\mathrm{Y_j} = 1 \mid \mathrm{X_j} = \mathrm{x_j})\mathrm{P}(\mathrm{S_j} = 1 \mid \mathrm{Y_j} = 1)}{\sum_{\mathrm{y'}} \mathrm{P}(\mathrm{Y_j} = \mathrm{y'} \mid \mathrm{X_j} = \mathrm{x_j})\,\mathrm{P}(\mathrm{S_j} = 1 \mid \mathrm{Y_j} = \mathrm{y'})}. \quad (2)$$

The second term of the numerator is equal under both sides. After dividing both sides by this term and rearranging, we obtain

$$\mathrm{P}(\mathrm{Y_i} = 1 \mid \mathrm{X_i} = \mathrm{x_i}) > P(\mathrm{Y_j} = 1 \mid \mathrm{X_j} = \mathrm{x_j}). \quad (3)$$

This completes the derivation. The derivation remains the same when considering an ensemble of classifiers with the exception that the estimated probability $\mathrm{P}(\mathrm{Y_j} = 1 \mid \mathrm{S_j} = 1, \mathrm{X_j} = \mathrm{x_j})$ is now computed by many different classifiers and then averaged.

The derivation above holds for a binary classifier, demonstrating that driver gene rankings are preserved under down-sampling. However, the derivation does not hold for TSG and OG rankings as the classifier is not binary in this scenario. Rather, a stronger set of assumptions is required to guarantee rank preservation. In the presence of more than two classes, gene i remains ranked above gene j when the following inequality holds:

$$\sum_{\mathrm{k'}} P_i^{\mathrm{k'}}\, \mathrm{P}(\mathrm{S_i} = 1 \mid \mathrm{Y_j} = \mathrm{k'}) > \sum_{\mathrm{k'}} P_j^{\mathrm{k'}}\, \mathrm{P}(\mathrm{S_j} = 1 \mid \mathrm{Y_j} = \mathrm{k'}), \quad (4)$$

where P_i^k is a shorthand notation for $\mathrm{P}(\mathrm{Y_i} = \mathrm{k} \mid \mathrm{X_i} = \mathrm{x_i})$. This inequality is likely to hold for pairs of genes with similar posterior probability distributions.

7) Assessing sensitivity to sample bias towards frequently altered driver genes

Infrequently altered driver genes are expected to often have weaker cancer-driving phenotypes than the well-known drivers used in the training set. As such, infrequent drivers have weaker tumor genomic signatures and potentially weaker cancer-associated non-genomic signatures, although exceptions are expected.

We applied a sample bias correction technique to evaluate the impact of the overrepresentation of frequently altered drivers in the high confidence set which was used for training. Although

several sample bias correction approaches have been previously proposed[19–21], they are not suitable for a PU learning setting wherein there is no clear distinction between the training and testing set. Instead, we propose a domain specific probabilistic model for this task.

Our probabilistic model treats the true (unknown) cancer role of a gene as a latent random variable and its training set label, which represents its currently known role, as an observed random variable. Below, we show that the classifier estimate of being a driver gene serves as a lower bound for the true estimated probability of being a driver gene. An upper bound on the estimated probability of being a true driver gene can also be derived, as detailed below.

We formalize the model as follows. For each gene i, we denote X_i as the vector of features of gene i. We further denote two random variables D_i and Y_i, which indicate the true and training set label of the gene, respectively. Formally, $D_i, Y_i \in \{TSG, OG, BG\}$, where the values indicate a tumor suppressor gene, an oncogene, and a background gene, respectively. We denote this model as the trinary model. It is also possible to define a binary model with only two values, DG, BG, where DG denotes a driver gene, such that the statements $D_i = DG$, $Y_i = DG$ are equivalent to the statements $D_i \in \{TSG, OG\}, Y_i \in \{TSG, OG\}$, respectively. The following derivation applies to both the trinary and binary models. In the following, we do not explicitly condition on the selection variable S_i defined in the previous section. Conditioning on this variable does not affect the derivations, because this variable is conditionally independent of all other variables given Y_i, thus it does not affect the independence structures described below.

We are interested in ranking genes according to the posterior probability being a TSG, OG or DG, $P(D_i = d \mid X_i = x_i)$ for $d \in \{TSG, OG, DG\}$. As this quantity cannot be estimated directly, we derive lower and upper bounds to be used for gene ranking. Assuming all training set driver gene labels are correct, for every $d \neq BG$ and for every features vector x_*, the following inequalities hold:

$$\mathrm{P}(\mathrm{Y_i} = \mathrm{d} \mid \mathrm{X_i} = \mathrm{x_i}) \leq \mathrm{P}(\mathrm{D_i} = \mathrm{d} \mid \mathrm{X_i} = \mathrm{x_i}) \leq \frac{\mathrm{P}(\mathrm{Y_i} = \mathrm{d} \mid \mathrm{X_i} = \mathrm{x_i})}{\mathrm{P}\left(\mathrm{Y_i} = \mathrm{d} \mid \widetilde{\mathrm{X}}_\mathrm{i} = \tilde{\mathrm{x}}_\mathrm{i}, \widehat{\mathrm{X}}_\mathrm{i} = \hat{x}_*\right)}. \quad (5)$$

Here, $\widetilde{X}_i$ consists of the tumor genomic features of gene *i*, $\widehat{\mathrm{X}}_\mathrm{i}$ is the complementary vector of all other features, and $\tilde{x}_i$, $\hat{x}_*$ consist of the corresponding entries of x_i and x_*, respectively. A more detailed definition is given below. To find the vector x_* yielding the tightest possible upper bound in Equation 5, we iterate over all vectors of training set driver genes (high confidence set) and use CARNAF to estimate the upper bound.

The lower bound reflects the fact that the training set driver genes are a subset of all existing drivers. The upper bound consists of a sample bias correction term, which increases the estimated probability of less frequently mutated genes, since such genes were less likely to be included among the training set driver genes (Supplementary Fig. 5). The denominator of the right hand side of Equation 5 encodes the probability for a synthetic gene, consisting of the tumor genomics features of gene *i* along with the non-tumor genomics features of a different gene. The upper bound in Equation 5 therefore assesses to what degree the probability

estimated by the classifier for gene *i* may have been larger had the gene contained different non-genomics features. If the upper bound is high for every tested vector $\hat{x}_*$ of non-genomic features, the gene may be underestimated due to weak tumor genomics features. In contrast, if the upper bound is low for at least one vector $\hat{x}_*$, it suggests that the tumor genomics features of this gene do not substantially hinder its detection and the resulting correction will be small.

7.1) Lower Bound

The lower bound of Equation 5 is derived under the assumptions that the high confidence driver gene set used for training does not contain non-driver genes and that all 165 high confidence genes are correctly identified as TSGs or OGs. These assumptions are encoded as the following pair of equations.

$$P(Y_i \neq BG, Y_i \neq D_i \mid D_i \neq BG, X_i = x_i) = 0. \quad (6)$$

$$P(Y_i \neq BG \mid D_i = BG, X_i = x_i) = 0. \quad (7)$$

Using the assumptions above, the law of total probability, and restricting $d \neq BG$, we have

$$P(Y_i = d \mid X_i = x_i) = P(D_i = d \mid X_i = x_i)P(Y_i = d \mid D_i = d, X_i = x_i). \quad (8)$$

We conclude that $P(D_i = d \mid X_i = x_i)$ is lower bounded by $P(Y_i = d \mid X_i = x_i)$ whenever $d \neq BG$. This completes the derivation of the lower bound of Equation 5.

7.2) Upper bound

To derive the upper bound of Equation 5, we first divide the vector X_i into two non-overlapping vectors, $X_i = (\tilde{X}_i, \hat{X}_i)$, such that $\tilde{X}_i$ consists of the tumor genomics features and $\hat{X}_i$ is the complementary vector of all other features. Thus, we obtain

$$P(Y_i = d \mid D_i = d, X_i = x_i) = P(Y_i = d \mid D_i = d, \tilde{X}_i = \tilde{x}_i). \quad (9)$$

Equation 9 encodes the assertion that Y_i is conditionally independent of $\hat{X}_i$ given D_i and $\tilde{X}_i$. This results from the approximation that the training set driver genes were discovered using tumor genomics features alone, and validated to ensure that the findings are genuine. Therefore, the non-tumor genomics features $\hat{X}_i$ did not directly participate in the creation of the label Y_i.

Next, we observe that for every features vector x_*, use of the conditional independence of Equation 9 along with Equation 8 yields

$$P(Y_i = d \mid \tilde{X}_i = \tilde{x}_i, \hat{X}_i = \hat{x}_*) = P(D_i = d \mid \tilde{X}_i = \tilde{x}_i, \hat{X}_i = \hat{x}_*)P(Y_i = d \mid D_i = d, \tilde{X}_i = \tilde{x}_i). \quad (10)$$

In the next step, we rearrange Equation 8 as follows.

$$P(D_i = d \mid X_i = x_i) = \frac{P(Y_i = d \mid X_i = x_i)}{P(Y_i = d \mid D_i = d, X_i = x_i)}. \quad (11)$$

Finally, we incorporate Equations 9 and 10 into the denominator of Equation 11 to obtain

$$P(D_i = d \mid X_i = x_i) = \frac{P(Y_i = d \mid X_i = x_i)}{P(Y_i = d \mid \tilde{X}_i = \tilde{x}_i, \hat{X}_i = \hat{x}_*)} P(D_i = d \mid \tilde{X}_i = \tilde{x}_i, \hat{X}_i = \hat{x}_*). \quad (12)$$

The upper bound of Equation 5 is obtained from Equation 12 by upper-bounding the last element of the right hand side by 1.0.

7.3) Assessing the sensitivity to sampling bias

We used Equation 5 to assess the sensitivity of CARNAF to sampling bias towards frequently altered driver genes by ranking genes according to the upper bound. Equation 12 demonstrates that this bound is likely to be tight for top ranked genes since the numerator of Equation 12 for such genes is large. Furthermore, the denominator is always larger than the numerator, and additionally the last element in Equation 12 is larger than the denominator owing to Equation 8. Consequently, all three quantities are large for top ranked genes, and the element on the right hand side is expected to be close to 1, yielding a tight bound.

Sensitivity to sampling bias was measured by measuring the overlap between top ranked gene lists, with and without applying the sampling bias correction (Supplementary Table 7). For completeness, we also report the overlap between these lists and the list of top ranked genes when using only non-tumor genomics features, as the latter list is expected to be less sensitive to sampling bias. The results show that all three methods yield similar lists of top ranked genes, indicating that sampling bias has a minor impact on top ranked genes.

References

1. Mishra, R. Glycogen synthase kinase 3 beta: can it be a target for oral cancer. *Mol. Cancer* **9,** 144 (2010).
2. Chen, H.-Z., Tsai, S.-Y. & Leone, G. Emerging roles of E2Fs in cancer: an exit from cell cycle control. *Nat. Rev. Cancer* **9,** 785–797 (2009).
3. Fang, Y. & Nicholl, M. B. A dual role for sirtuin 1 in tumorigenesis. *Curr. Pharm. Des.* **20,** 2634–2636 (2014).
4. Han, J. *et al.* Analysis of 41 cancer cell lines reveals excessive allelic loss and novel mutations in the SIRT1 gene. *Cell Cycle Georget. Tex* **12,** 263–270 (2013).
5. Wang, Y., Qi, X., Wang, F., Jiang, J. & Guo, Q. Association between TGFBR1 polymorphisms and cancer risk: a meta-analysis of 35 case-control studies. *PloS One* **7,** e42899 (2012).
6. Lin, Z. & Fang, D. The Roles of SIRT1 in Cancer. *Genes Cancer* **4,** 97–104 (2013).
7. Johnson, N. *et al.* Compromised CDK1 activity sensitizes BRCA-proficient cancers to PARP inhibition. *Nat. Med.* **17,** 875–882 (2011).
8. Kang, J., Sergio, C. M., Sutherland, R. L. & Musgrove, E. A. Targeting cyclin-dependent kinase 1 (CDK1) but not CDK4/6 or CDK2 is selectively lethal to MYC-dependent human breast cancer cells. *BMC Cancer* **14,** 32 (2014).
9. Sung, W.-W. *et al.* High nuclear/cytoplasmic ratio of Cdk1 expression predicts poor prognosis in colorectal cancer patients. *BMC Cancer* **14,** 951 (2014).
10. Zeestraten, E. C. M. *et al.* Specific activity of cyclin-dependent kinase I is a new potential predictor of tumour recurrence in stage II colon cancer. *Br. J. Cancer* **106,** 133–140 (2012).
11. Liu, C.-W. *et al.* Snail regulates Nanog status during the epithelial-mesenchymal transition via the Smad1/Akt/GSK3β signaling pathway in non-small-cell lung cancer. *Oncotarget* **5,** 3880–3894 (2014).
12. Katsuno, Y. *et al.* Bone morphogenetic protein signaling enhances invasion and bone metastasis of breast cancer cells through Smad pathway. *Oncogene* **27,** 6322–6333 (2008).
13. Lawrence, M. S. *et al.* Mutational heterogeneity in cancer and the search for new cancer-associated genes. *Nature* **499,** 214–218 (2013).
14. Davoli, T. *et al.* Cumulative haploinsufficiency and triplosensitivity drive aneuploidy patterns and shape the cancer genome. *Cell* **155,** 948–962 (2013).
15. Beroukhim, R. *et al.* The landscape of somatic copy-number alteration across human cancers. *Nature* **463,** 899–905 (2010).
16. Georgi, B., Voight, B. F. & Bućan, M. From mouse to human: evolutionary genomics analysis of human orthologs of essential genes. *PLoS Genet.* **9,** e1003484 (2013).
17. Ouedraogo, M. *et al.* The Duplicated Genes Database: Identification and Functional Annotation of Co-Localised Duplicated Genes across Genomes. *PLoS ONE* **7,** e50653 (2012).
18. Leiserson, M. D. M. *et al.* Pan-cancer network analysis identifies combinations of rare somatic mutations across pathways and protein complexes. *Nat. Genet.* **47,** 106–114 (2015).
19. Huang, J., Gretton, A., Borgwardt, K. M., Schölkopf, B. & Smola, A. J. Correcting sample selection bias by unlabeled data. in *Advances in neural information processing systems* 601–608 (2006).

20. Zadrozny, B. Learning and evaluating classifiers under sample selection bias. in *Proceedings of the twenty-first international conference on Machine learning* 114 (ACM, 2004).
21. Sugiyama, M., Nakajima, S., Kashima, H., Buenau, P. V. & Kawanabe, M. Direct importance estimation with model selection and its application to covariate shift adaptation. in *Advances in neural information processing systems* 1433–1440 (2008).
22. Barretina, J. *et al.* The Cancer Cell Line Encyclopedia enables predictive modelling of anticancer drug sensitivity. *Nature* **483,** 603–607 (2012).

Supplementary Figures

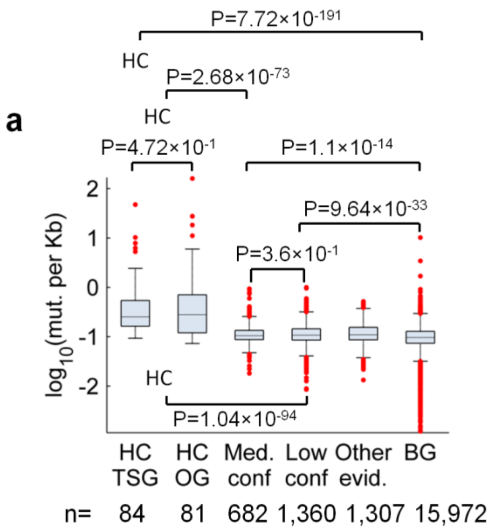

Figure S1: **Known driver genes are frequently mutated.** (a) Comparison of mutation frequency encoded by high confidence (HC) TSGs, high confidence OGs, medium confidence drivers, low confidence genes, other evidence genes that are present in at least one data source used in this work (Online Methods), and background genes (BGs). The numbers were computed by summing the number of mutations in COSMIC v.69 and dividing by the gene length. P values derived using the Welch t-test. Lines with the word "HC" on the left indicate a comparison between high confidence driver genes (TSGs and OGs combined) with another group. Genes known to act as drivers have a high mutation rate. (b) The same data as in panel (a) but without the log-scale (some outliers are outside of the visible scale). Percentiles were calculated in the absence of outliers, thus bar percentile values may slightly differ between the panels due to changes in values that are considered outliers following log transformation

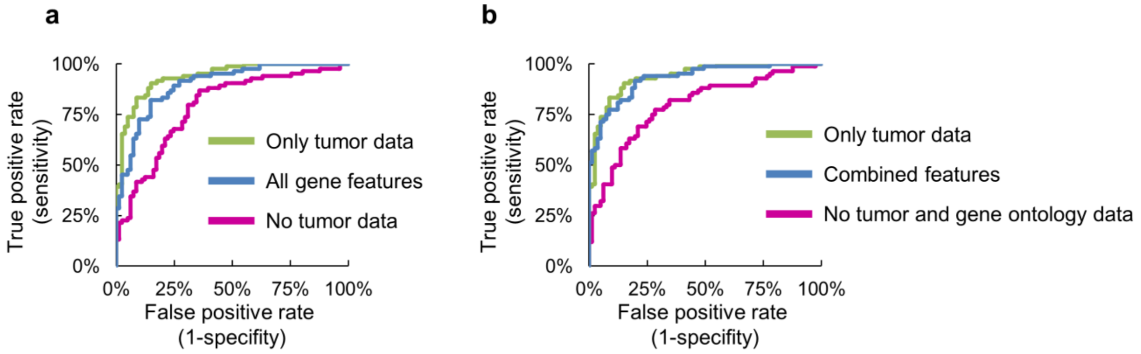

Figure S2: Driver gene classification accuracy as TSG or OG. The Receiver Operating Characteristic (ROC) curve is presented for classification of the high confidence driver genes as TSGs (n=84) or OGs (n=81). (a) ROC curve for CARNAF predictions. (b) ROC curve for CARNAF predictions performed without gene ontology features in order to reduce bias towards well-studied genes.

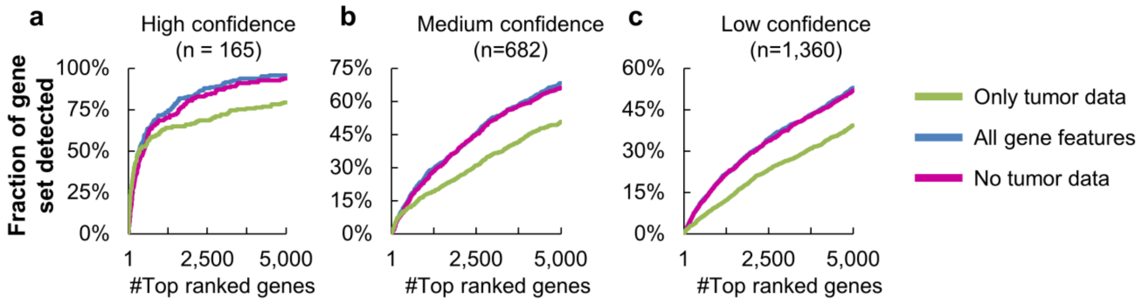

Figure S3: The cumulative rate of driver gene detection is enhanced by non-tumor genomics features. The cumulative detection rate is shown for three gene sets: (a) high confidence driver genes, (b) medium confidence genes, and (c) low confidence genes. The cumulative detection rate refers to the fraction of genes detected at the corresponding gene rank. Going from left to right, the genes considered in each panel are excluded from subsequent panels.

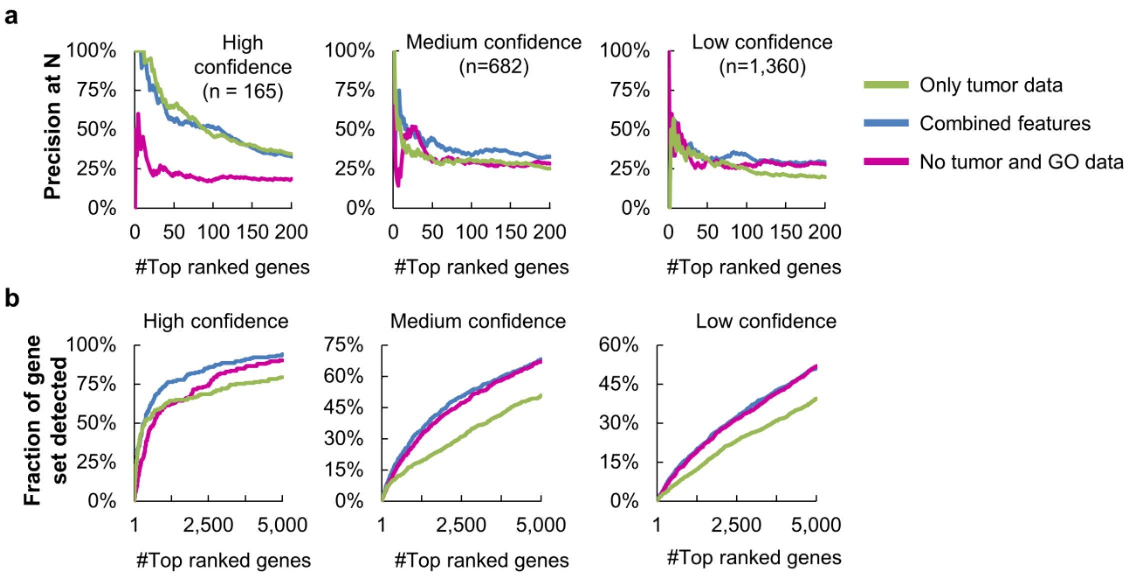

Figure S4: Driver gene detection performance without gene ontology features. CARNAF performance is depicted with omission of gene ontology features to reduce bias towards well-studied genes. (a) Precision and (b) cumulative detection rate is shown for three gene sets: high confidence driver genes, medium confidence genes, and low confidence genes. Precision in this scenario is equivalent to the fraction of detected genes. The cumulative detection rate refers to the fraction of genes detected at the corresponding gene rank. Going from left to right, the genes considered in each panel are excluded from subsequent panels.

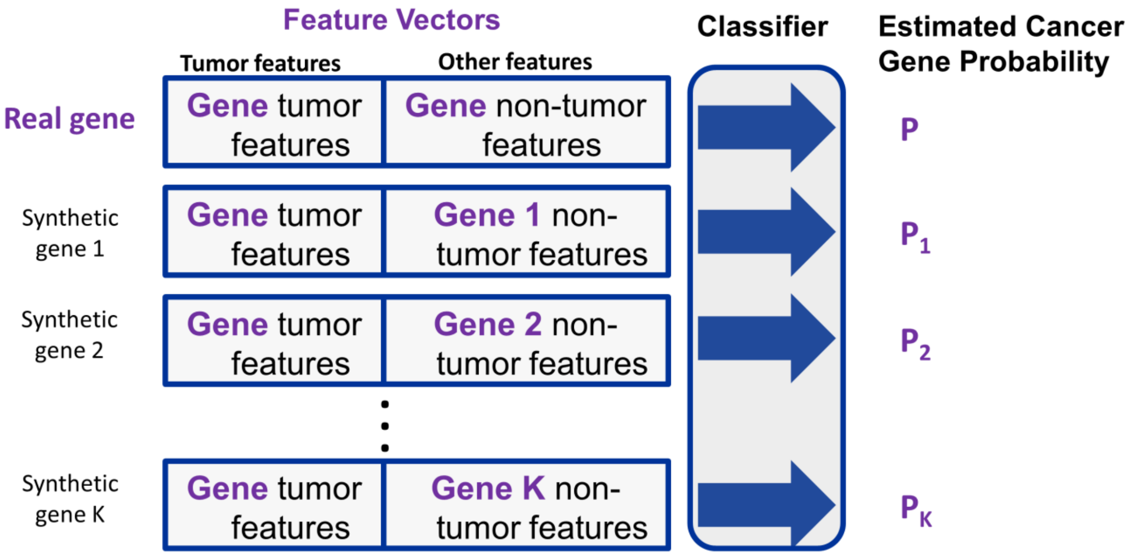

Figure S5: Driver gene upper bound probability derivation using synthetic feature vectors. K synthetic feature vectors, one for each high confidence driver, were constructed for each gene. Each synthetic feature vector contains the tumor features of the gene of interest along with the non-tumor features of one of the high confidence driver genes. The cancer gene probability of each feature vector was estimated by CARNAF. The upper bound is defined as P/P_i, where P is the estimated probability for the tested gene and P_i is the largest estimated probability for a synthetic feature vector.

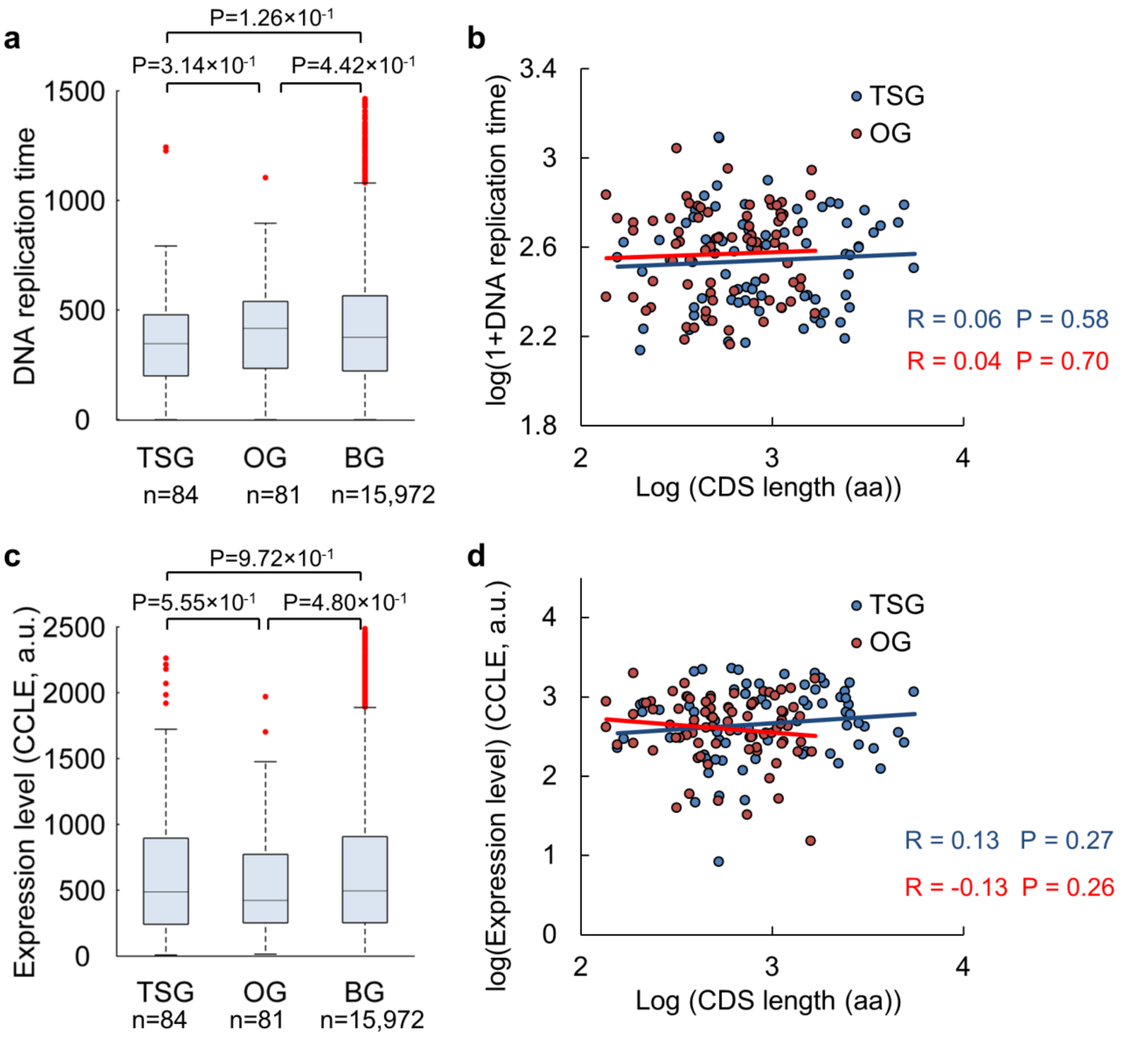

Figure S6: Driver gene protein size is not associated with DNA replication time or gene expression levels in cell lines. (a) Comparison of DNA replication times for TSGs, OGs, and background genes (BGs). (b) Log-log plot of DNA replication time against protein size shows little association for both TSGs and OGs. (c) Comparison of TSG, OG, and BG gene expression levels from cell lines in the Cancer Cell Line Encyclopedia (CCLE)[22]. (d) Log-log plot of expression levels against protein size shows little association for both TSGs and OGs. P values in (a,c) are derived using the KS test.

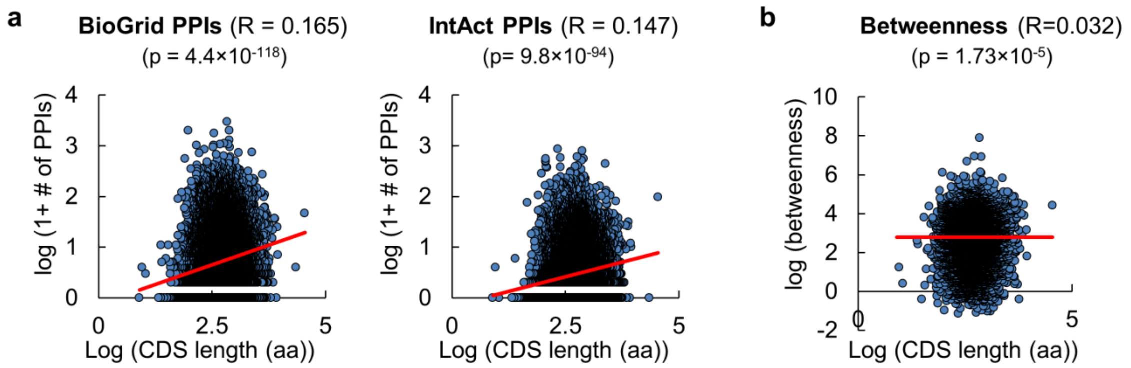

Figure S7: Genome-wide association between network centrality and protein size. (a) Log-log plot of number of protein-protein interactions versus protein size shows positive association between the two gene properties. (b) Log-log plot of betweenness values (presence in shortest paths in the BioGrid protein network) versus protein size shows a mild negative association between the two gene properties.

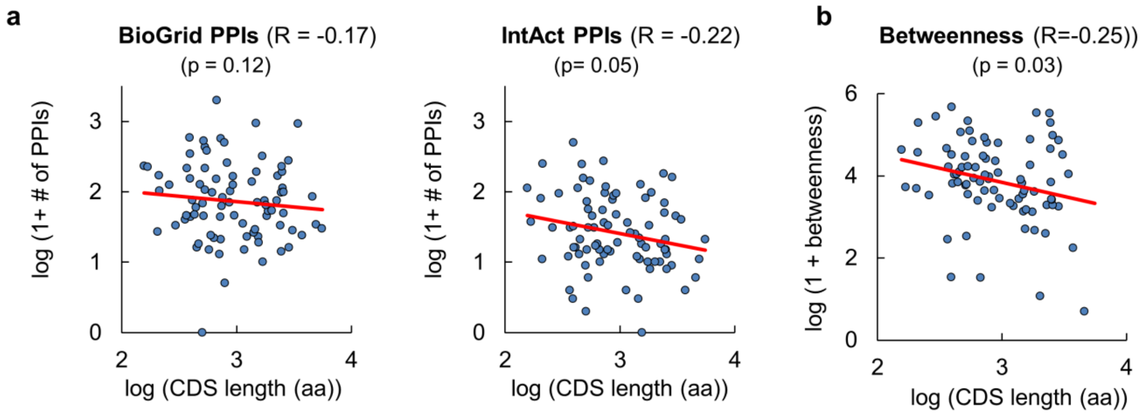

Figure S8: TSG protein size is not positively associated with network centrality or connectivity. (a) Log-log plot of number of protein-protein interactions versus protein size for TSGs across three data sources. (b) Log-log plot of betweenness values (presence in shortest paths in the BioGrid protein network) versus protein size.

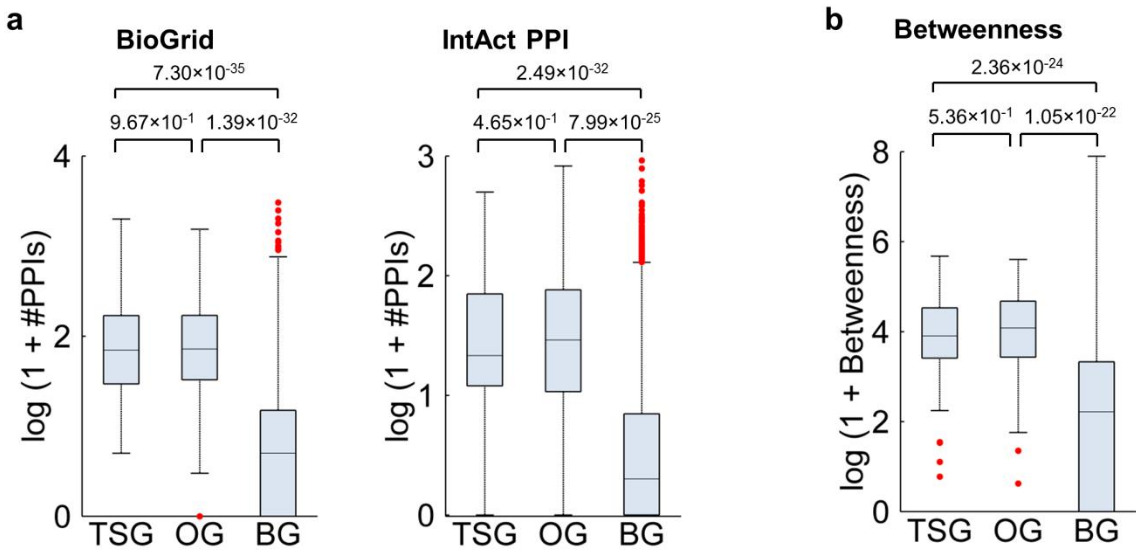

Figure S9: TSGs and OGs have similar network centrality and connectivity. (a) Comparison of number of protein-protein interactions (PPIs) per gene across two PPI data sources. (b) Comparison of betweenness values (presence in shortest paths in the BioGrid protein network). All P values are derived using the Kolmogorov-Smirnov test and shown above the corresponding boxes.

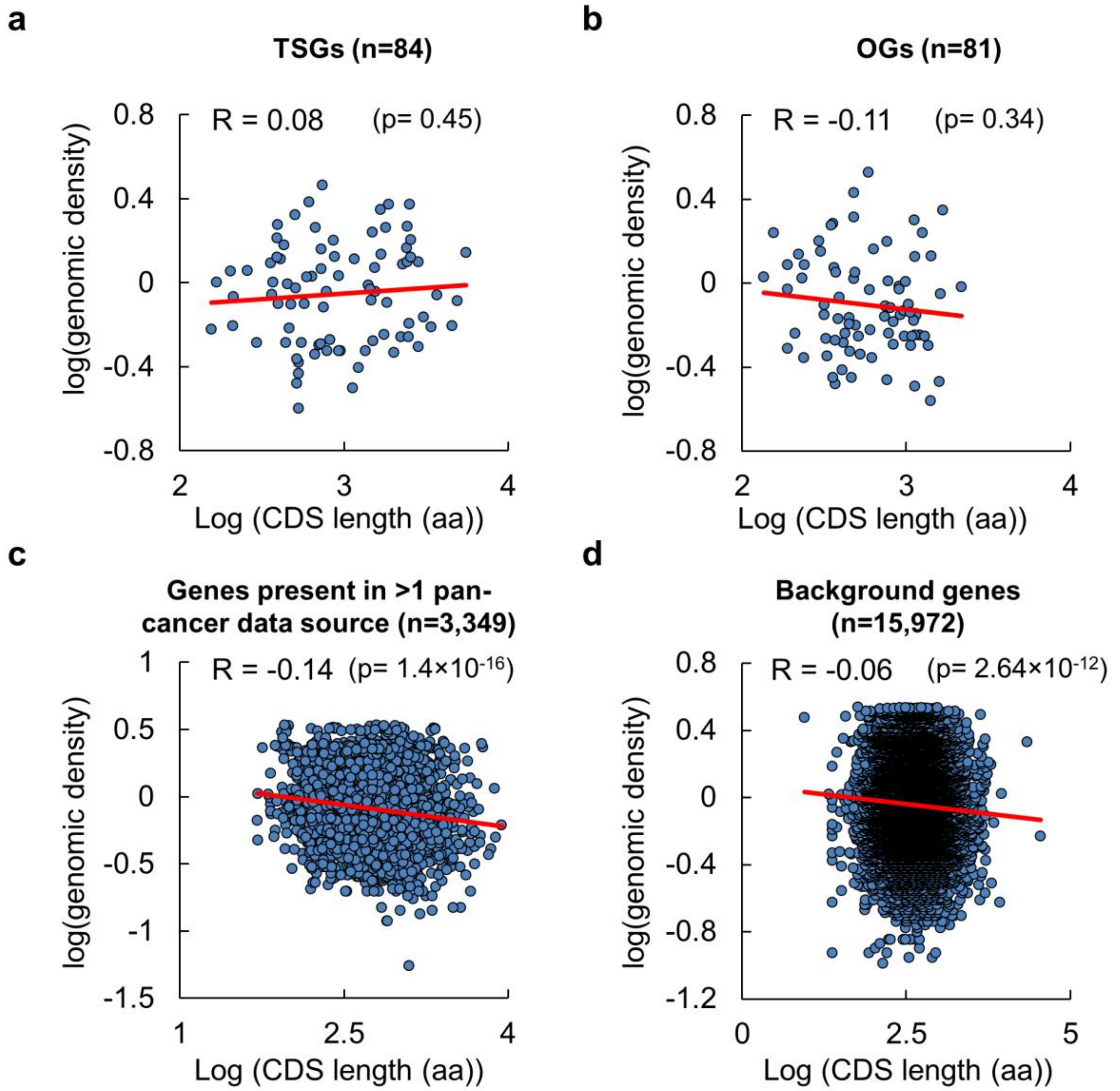

Figure S10: Protein size is weakly associated with gene density for both driver and non-driver genes. Log gene density is plotted against log protein size (aa) for (a) high confidence TSGs, (b) high confidence OGs, (c) genes that are not in the high confidence and are present in at least 1 data source, and (d) background genes

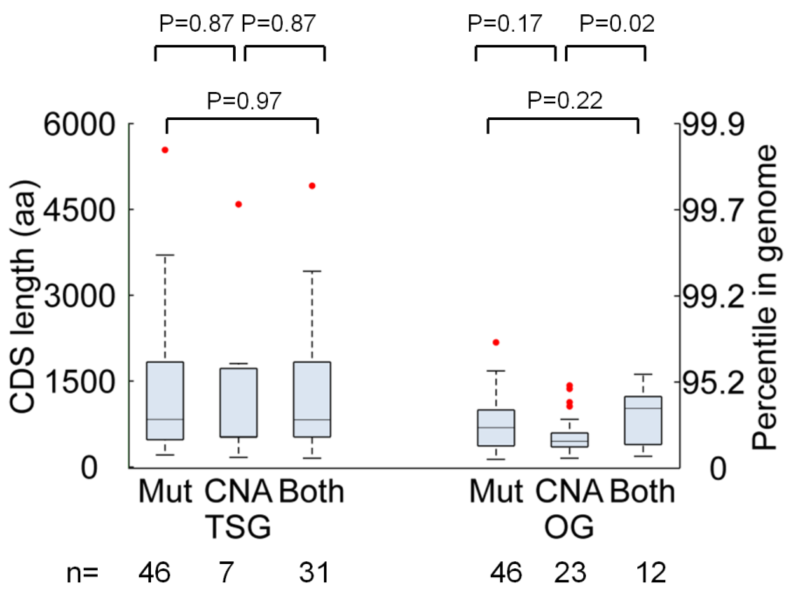

Figure S11: TSG mode of inactivation is not associated with TSG protein size. Comparison of protein size across different modes of inactivation and activation shows similar size distributions across TSG mode of inactivation. CNA – copy number alteration (deletions or gains)

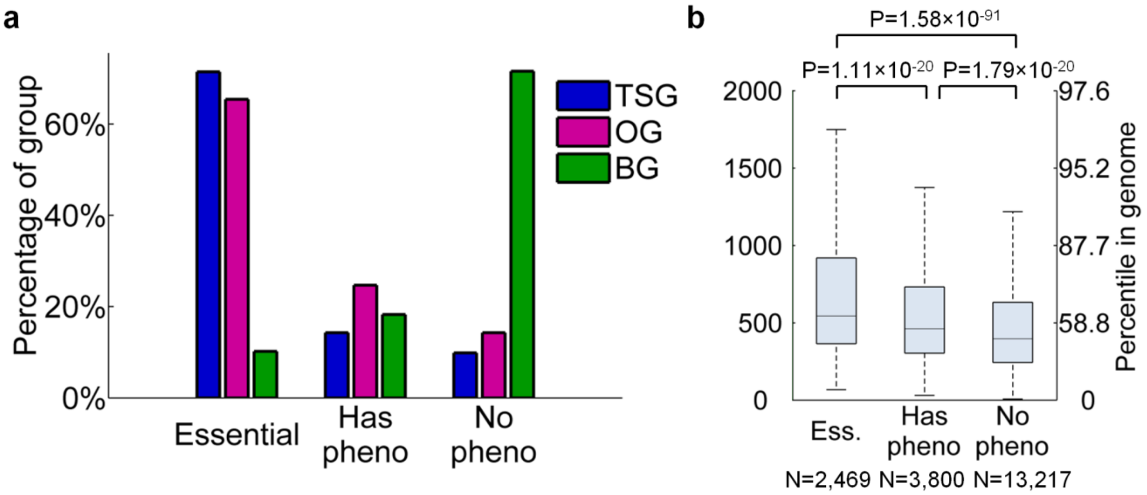

Figure S12: TSGs and OGs have similar fractions of essential genes, which tend to encode large proteins. (a) Depiction of the fraction of TSGs, OGs, and BGs (background genes) that reside in one of three categories as predicted by mouse homology[16]: essential genes, genes whose loss results in a measurable phenotype but are not essential, and genes with no phenotype. (b) Essential genes encode large proteins compared to non-essential genes. Outliers not shown due to large abundance and to reduce y-axis scale range.

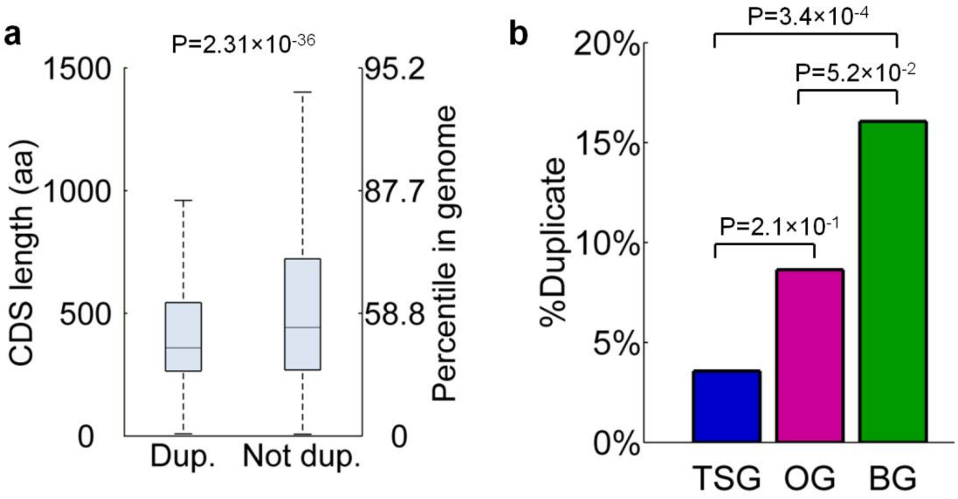

Figure S13: Duplicate genes encode small proteins and driver genes have low duplication rates. (a) The distribution of duplicate gene protein sizes is shifted towards smaller proteins compared to non-duplicate genes. Outliers not shown due to large abundance and to reduce y-axis scale range. P values derived using the Welch t-test. (b) Comparison of gene duplication rates shows that driver genes have low duplication rates, but the difference between TSGs and OGs is not substantial. P values are derived using Fisher's exact test.

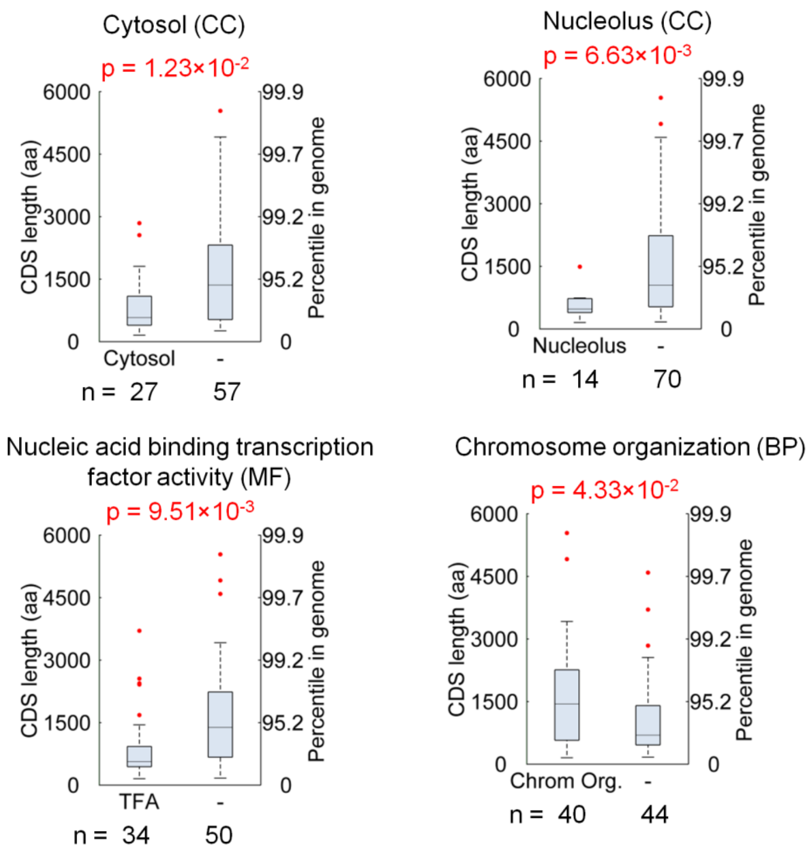

Figure S14: Coding sequence length distributions for TSGs are shown according to GO slim annotations. The four GO slim annotations with t-test $p<0.05$ of being different from the remaining TSGs are shown (after Bonferonni correction, the 5% significance cutoff is 0.00079). Large TSG proteins are frequently involved in chromosome organization processes.

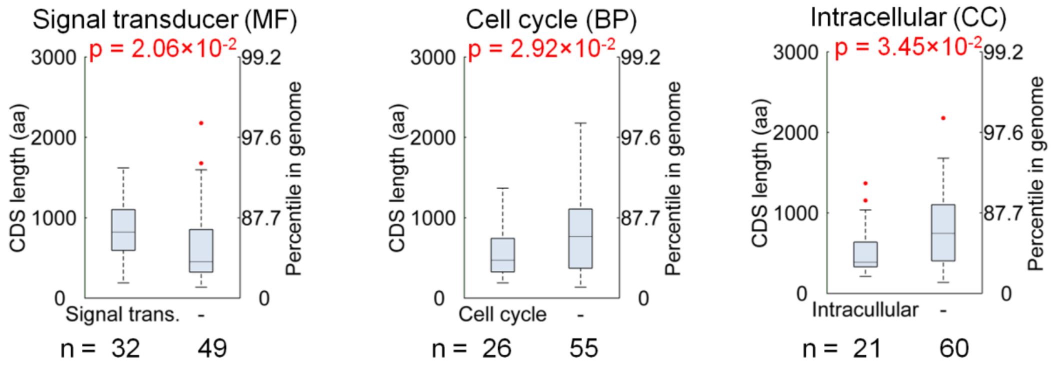

Figure S15: Coding sequence length distributions for OGs are shown according to GO slim annotations. The three GO slim annotations with t-test p-value <0.05 of being different from the remaining OGs are shown (after Bonferonni correction, the 5% significance cutoff is 0.00082).

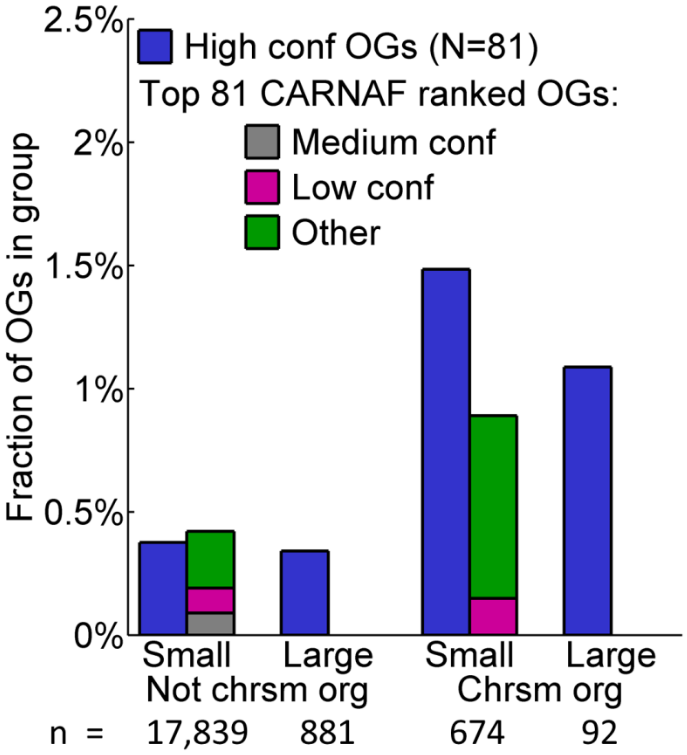

Figure S16: OGs distribution according to protein size and role in chromosome organization processes. This figure is formatted as Fig. 4a, with differences in y-axis scaling. The abundance of high confidence OGs and CARNAF predicted OGs (excluding high confidence drivers) encoding very large (top 5% in genome) and small (the remaining 95%) proteins with respect to participation in chromosome organization processes. The top 81 CARNAF OG predictions were selected to match the abundance of OGs in the high confidence set (n=81). CARNAF predictions that overlap with the medium and low confidence driver gene sets are shown. Chrsm refers to the gene ontology biological process annotation chromosome organization.

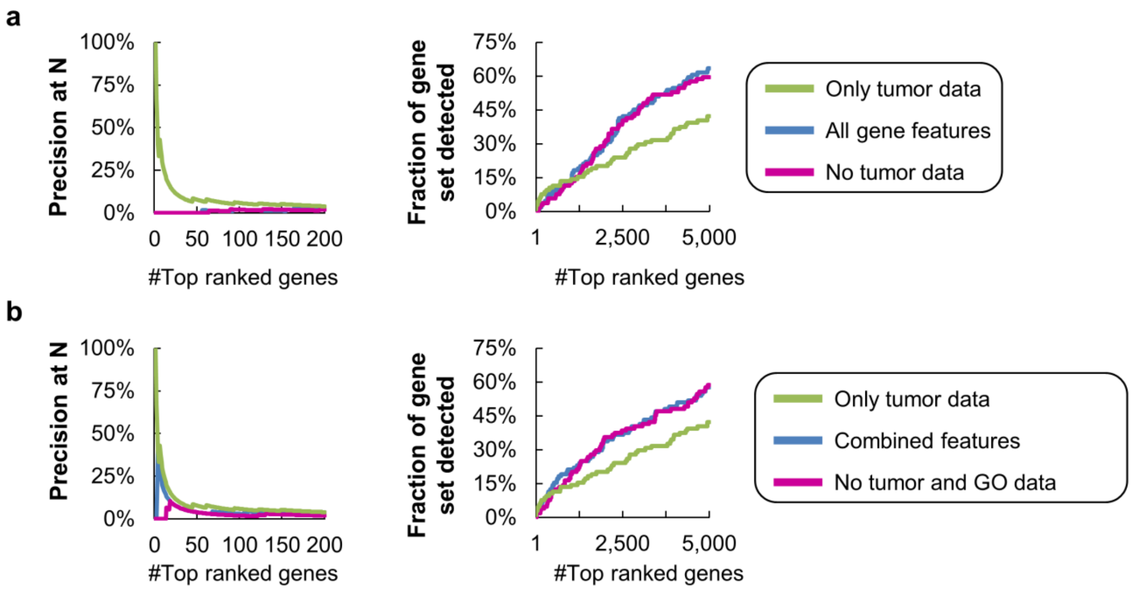

Figure S17: Comparison of CARNAF predictions with HotNet2 detected genes. Precision at N and cumulative detection rate of CARNAF are presented with respect to the 104 HotNet2 genes not present in the high confidence driver gene set. (a) Results using CARNAF rankings with all features. (b) Results obtained when excluding gene ontology features to reduce bias towards well-studied genes. Precision in this scenario is equivalent to the fraction of detected genes. The cumulative detection rate refers to the fraction of genes detected at the corresponding gene rank.

Supplementary Tables

Table Number	Title	Location
Supplementary Table 1	Features used in CARNAF	Excel file
Supplementary Table 2	Features used in study, but not in CARNAF	Excel file
Supplementary Table 3	Driver gene data sources used in study	Supplementary document
Supplementary Table 4	Genes present in 15 multi-tumor type data sources	Excel file
Supplementary Table 5	CARNAF gene ranks	Excel file
Supplementary Table 6	Evaluating the sensitivity of CARNAF to specific training set choices	Supplementary document
Supplementary Table 7	Evaluating the effect of sampling bias on CARNAF gene rankings	Supplementary document
Supplementary Table 8	Curation of top 15 CARNAF driver gene predictions not included in training set drivers	Excel file
Supplementary Table 9	Feature importance	Supplementary document
Supplementary Table 10	Feature association for high confidence TSG vs high confidence OG labels	Supplementary document
Supplementary Table 11	Feature association for high confidence driver genes vs background genes (BGs)	Supplementary document
Supplementary Table 12	Comparison of TSG and OG protein size versus non-TSG/OG protein size for GO slim categories and according to expressed tissues	Excel file
Supplementary Table 13	Comparison of TSG protein size versus non-TSG protein size for lower level chromosome organization GO categories	Excel file

Supplementary Table 3. Driver gene data sources used in study. 15 multi-tumor type data sources were compiled to aid in CARNAF training and result evaluation. Gene label annotations included both mutation and copy number based driver genes. Several sources included functional annotation as TSGs and OGs. Translocations were not considered.

**Data Source**	Gene labels	**Description**
TUSON explorer[1]	Mutation-based TSGs and OGs	A statistical approach that uses sequenced tumors to classify genes as TSGs or OGs using four mutation pattern features.
Vogelstein *et al.*[2]	Mutation and copy number alteration based TSGs and OGs	A highly-cited cancer genomics review that classified well known driver genes as TSGs and OGs using manual curation and the 20/20 rule. The rule classifies drivers with at least 20% inactivating mutations as TSGs, and those with at least 20% of mutations clustered at specific amino acids as OGs. The rule has a few exceptions.
Zack *et al.*[3]	Amplified and deleted genes in cancer	Statistical analysis of small copy number alterations from a set of approximately 5,000 tumors. The authors manually curated amplified and deleted genes for known evidence.
CGC[4]	Mutated and copy number alteration based driver genes	A highly-referenced manual curation of driver genes in cancer. Version 69 was used.
Garnett *et al.*[5]	Expression-based gene biomarkers to drugs	A large cell line-based screen to discover cancer drug biomarkers that tested 130 drugs across ~600 cell lines. Expression was measured for 14,500 genes. Genes with absolute effect >3 were selected from Supplementary Table 3.
Santarius *et al*.[6]	Amplification-based driver genes	A rigorous study using clinical, experimental, and biological knowledge evidence to identify amplification and overexpression based driver genes in cancer.
Lawrence *et al.*[7]	Mutated cancer genes	Statistical analysis for significantly mutated cancer genes using approximately 5,000 tumors. The method corrects for known factors that affect mutation rates, notably expression levels and DNA replication time. Genes were taken from the union the high-confidence list (n=219). Genes marked as 'discussed' were those in which the authors directly or indirectly suggested a TSG or OG role in the main text.
2020 genome-wide implementation	Mutation-based TSGs and OGs	Genome-wide implementation by the authors of the 20/20 rule as described previously[2] using Cosmic tumor data (v69). The original 20/20 rule was applied on 138 driver genes.
Tag DB[8]	TSGs and OGs	A text mining approach to identify TSGs and OGs.
TSGene[9]	TSGs	A literature curation effort to compile a large list of TSGs.
ActiveDriver[10]	Mutated cancer genes	A method that identifies genes enriched in phosphorylation site-associated mutations. Genes were retrieved from method implementation on 3,205 tumors across 12 cancer types[11].
OncoDriveFM[12]	Mutated cancer genes	A method that detects genes with a bias towards high functional mutations as predicted using three independent tools. Genes were retrieved from method implementation on 3,205 tumors across 12 cancer types[11].
OncoDriveClust[13]	Mutated cancer genes	A method that identifies genes that contain high regional clustering of mutations. Genes were retrieved from method implementation on 3,205 tumors across 12 cancer types[11].
MuSiC[14]	Mutated cancer genes	A study that searched for genes mutated more frequently than expected. Genes were retrieved from method implementation on 3,205 tumors across 12 cancer types[11].
HotNet2[15]	Mutated cancer genes	A study that used protein-protein network knowledge in conjunction with mutation data from 3,000 tumors to search for infrequently mutated driver genes.

Supplementary Table 3 references

1. Davoli, T. *et al.* Cumulative haploinsufficiency and triplosensitivity drive aneuploidy patterns and shape the cancer genome. *Cell* **155,** 948–962 (2013).
2. Vogelstein, B. *et al.* Cancer Genome Landscapes. *Science* **339,** 1546–1558 (2013).
3. Zack, T. I. *et al.* Pan-cancer patterns of somatic copy number alteration. *Nat. Genet.* **45,** 1134–1140 (2013).
4. Futreal, P. A. *et al.* A census of human cancer genes. *Nat. Rev. Cancer* **4,** 177–183 (2004).
5. Garnett, M. J. *et al.* Systematic identification of genomic markers of drug sensitivity in cancer cells. *Nature* **483,** 570–575 (2012).
6. Santarius, T., Shipley, J., Brewer, D., Stratton, M. R. & Cooper, C. S. A census of amplified and overexpressed human cancer genes. *Nat. Rev. Cancer* **10,** 59–64 (2010).
7. Lawrence, M. S. *et al.* Discovery and saturation analysis of cancer genes across 21 tumour types. *Nature* **505,** 495–501 (2014).
8. Chen, J.-S., Hung, W.-S., Chan, H.-H., Tsai, S.-J. & Sun, H. S. In silico identification of oncogenic potential of fyn-related kinase in hepatocellular carcinoma. *Bioinforma. Oxf. Engl.* **29,** 420–427 (2013).
9. Zhao, M., Sun, J. & Zhao, Z. TSGene: a web resource for tumor suppressor genes. *Nucleic Acids Res.* **41,** D970–976 (2013).
10. Reimand, J. & Bader, G. D. Systematic analysis of somatic mutations in phosphorylation signaling predicts novel cancer drivers. *Mol. Syst. Biol.* **9,** 637 (2013).
11. Tamborero, D. *et al.* Comprehensive identification of mutational cancer driver genes across 12 tumor types. *Sci. Rep.* **3,** 2650 (2013).
12. Gonzalez-Perez, A. & Lopez-Bigas, N. Functional impact bias reveals cancer drivers. *Nucleic Acids Res.* **40,** e169 (2012).
13. Tamborero, D., Gonzalez-Perez, A. & Lopez-Bigas, N. OncodriveCLUST: exploiting the positional clustering of somatic mutations to identify cancer genes. *Bioinforma. Oxf. Engl.* **29,** 2238–2244 (2013).
14. Dees, N. D. *et al.* MuSiC: identifying mutational significance in cancer genomes. *Genome Res.* **22,** 1589–1598 (2012).
15. Leiserson, M. D. M. *et al.* Pan-cancer network analysis identifies combinations of rare somatic mutations across pathways and protein complexes. *Nat. Genet.* **47,** 106–114 (2015).

Supplementary Table 6. **Evaluating the sensitivity of CARNAF to the choice of training set.** The table reports the results of CARNAF when using slightly different training sets. In each of the 10 experiments, a random subset of 10% of the high confidence driver genes were removed from the training set. For each experiment, we measured how many genes (excluding the 165 high confidence driver genes) ranked as the top N most likely driver genes overlapped with the top N ranked genes of the original run of CARNAF. This overlap comparison was assessed for N=10, 25, 50, 100, 250, 500 genes. The results indicate that CARNAF is highly robust to the choice of specific training set used.

N	Exp. 1	Exp. 2	Exp. 3	Exp. 4	Exp. 5	Exp. 6	Exp. 7	Exp. 8	Exp. 9	Exp. 10
10	10	8	8	10	10	9	8	8	9	10
25	21	22	22	21	22	23	22	22	21	23
50	45	43	45	46	47	46	44	44	46	45
100	90	85	86	89	87	88	85	87	87	87
250	232	227	228	225	226	230	228	226	231	229
500	476	460	452	456	463	465	462	464	469	457

Supplementary Table 7. **Evaluating the effect of sampling bias on CARNAF gene rankings.** The table shows the number of shared genes that appear at the top CARNAF ranked lists created using all gene features (All tumor features) or all gene features excluding tumor genomics (No tumor features). LB and UB denote using the lower and upper bounds in Equation 5 in the Supplementary Note, respectively. The high intersection shows that the rankings are similar with both methods.

**Number of top ranked genes**	**All tumor features (LB ∩ UB)**	**No tumor features ∩ All tumor features (UB)**	**All tumor features (LB) ∩ No tumor features**
10	6	9	5
20	16	18	14
30	23	27	21
40	35	35	30
50	43	45	40
60	53	55	50
70	61	63	56
80	71	74	67
90	81	81	75
100	77	89	79

Supplementary Table 9. Feature importance. Top 15 binary (upper part) and top 15 continuous features (bottom) are shown. Importance measures for binary and continuous features use different scales, and thus cannot be directly compared[1].

Feature	Importance
Anatomical structure development (GO BP)	2.52
Nucleus (GO CC)	2.16
Signal transduction (GO BP)	2.10
Cell differentiation (GO BP)	1.99
Chromosome organization (GO BP)	1.91
Cell proliferation (GO BP)	1.89
Biosynthetic process (GO BP)	1.81
DNA binding (GO MF)	1.79
Cellular protein modification process (GO BP)	1.77
Cellular nitrogen compound metabolic process (GO BP)	1.69
Molecular function (GO MF)	1.68
Cell death (GO BP)	1.62
Cell cycle (GO BP)	1.39
Response to stress (GO BP)	1.38
Kinase activity (GO MF)	1.27
Tumor genomics: ratio of LOF to benign mutations	6.74
Number of PPIs	5.23
Number of GO slim terms	4.55
Haploinsufficiency (predicted)	4.44
Number of GO slim terms – biological process	4.10
Number of GO slim terms – molecular function	3.76
Tumor genomics: Entropy (mutation clustering vs random distribution)	3.67
Betweenness (presence in shortest paths in protein network)	3.63
Gene essentiality (predicted based on mouse homology)	3.41
Tumor genomics: ratio of splice site to benign mutations	3.28
Tumor genomics: ratio of damaging missense (predicted) to benign mutations	3.26
CDS length	3.11
Number of phosphoserines per residue	2.75
Number of phosphorylations per residue	2.71
Number of phosphotyrosines per residue	2.34

References

1. Hothorn, T., Hornik, K. & Zeileis, A. Unbiased recursive partitioning: A conditional inference framework. *J. Comput. Graph. Stat.* **15,** 651–674 (2006).

Supplementary Table 10. Feature association for high confidence TSG vs high confidence OG labels. The top 10 associated binary (top) and continuous features (bottom) are shown. Binary feature P values are derived by a Chi squared test. Continuous feature P values are derived using a Kolmogorov-Smirnov test. Expression values are arbitrary units. The 5% significance cutoff after Bonferonni correction is 0.000265.

**Feature**	**TSG**	**OG**	**P-value**
**Binary Features**	**% of genes**	**% of genes**	
Chromosome organization (GO BP)	47.6%	13.6%	2.25×10^{-6}
Signal transducer activity (GO MF)	16.7%	39.5%	1.07×10^{-3}
Nucleus (GO CC)	83.3%	63.0%	3.10×10^{-3}
Locomotion (GO BP)	3.6%	17.3%	3.77×10^{-3}
Plasma membrane (GO CC)	23.8%	44.4%	5.13×10^{-3}
Cellular component (GO CC)	17.9%	37.0%	5.68×10^{-3}
DNA binding (GO MF)	63.1%	42.0%	6.60×10^{-3}
Circulatory system process (GO BP)	0.0%	6.2%	2.08×10^{-2}
Vesicle-mediated transport (GO BP)	3.6%	13.6%	2.11×10^{-2}
Helicase activity (GO MF)	6.0%	0.0%	2.58×10^{-2}
**Continuous Features**	**Mean± s.d.**	**Mean± s.d.**	
Tumor mutation ratio of LOF to benign mutations	1.53± 1.70	0.17± 0.15	4.77×10^{-21}
Tumor mutation ratio of splice site to benign mutations	0.195± 0.289	0.030± 0.047	4.35×10^{-10}
Expression – Placenta	86± 581.43	49± 122	6.07×10^{-6}
Tumor genomics: gene copy gain frequency	0.70± 0.42	0.39± 0.47	1.16×10^{-5}
Number of phosphotyrosines per residue	0.0048± 0.0051	0.0085± 0.0076	1.86×10^{-4}
CDS length (amino acids)	1303± 1124	703± 433	3.04×10^{-4}
Expression – Lung	156± 1205	35± 66	7.85×10^{-4}
Tumor mutation entropy (randomness of distribution)	0.07± 0.14	0.26± 0.48	1.06×10^{-3}
Expression - Gall bladder	151± 995	44± 99	1.56×10^{-3}
Expression - Thyroid	99± 512	75± 288	1.60×10^{-3}

Supplementary Table 11. Feature association for high confidence driver genes vs background genes (BGs). P-values are shown for high confidence (HC) driver genes (both TSGs and OGs) compared to BGs. BGs are genes not present in any of the 15 multi-tumor type lists used in this study to compile driver genes (Online Methods). The top 10 associated binary (top) and continuous features (bottom) are shown. Binary feature P values are derived by a Chi squared test. Continuous feature P values are derived using a Kolmogorov-Smirnov test. The 5% significance cutoff after Bonferonni correction is 0.000242.

Feature	BGs	HC drivers	P-value
**Binary Features**	**% of genes**	**% of genes**	
Cell proliferation (GO BP)	6.3%	49.1%	1.05×10^{-104}
Chromosome organization (GO BP)	3.0%	30.9%	6.44×10^{-88}
Embryo development (GO BP)	3.9%	35.2%	1.18×10^{-87}
Anatomical structure development (GO BP)	15.2%	70.9%	2.79×10^{-85}
Cell death (GO BP)	7.4%	48.5%	4.58×10^{-85}
Cell differentiation (GO BP)	12.3%	61.8%	5.92×10^{-80}
Cell cycle (GO BP)	5.4%	39.4%	3.23×10^{-78}
Cellular protein modification process (GO BP)	12.8%	58.2%	1.62×10^{-65}
Kinase activity (GO MF)	5.7%	37.0%	8.44×10^{-64}
Enzyme binding (GO MF)	4.8%	33.3%	1.16×10^{-61}
**Continuous Features**	**Mean± s.d.**	**Mean± s.d.**	
Number of protein-protein interactions	12.0± 37.0	109.2± 170.9	1.03×10^{-64}
Number of GO slim terms	7.46± 6.20	22.26± 10.69	2.56×10^{-60}
Number of GO slim terms – molecular function	1.77± 1.57	4.71± 2.23	7.81×10^{-54}
Number of GO slim terms - biological process	3.45± 3.86	12.81± 7.24	1.14×10^{-52}
Gene essentiality (predicted based on mouse homology)[1]	0.39± 0.66	1.56± 0.70	1.75×10^{-51}
Haploinsufficiency (predicted)	0.27± 0.24	0.67± 0.30	6.14×10^{-45}
Number of GO slim terms - cellular component	2.24± 1.87	4.75± 2.67	1.09×10^{-30}
Betweenness (presence in protein network paths, a.u.)	213.7± 4,107	11.01± 25.30	1.15×10^{-29}
Tumors: Damaging missense to benign mutation ratio[2]	0.72± 0.37	1.62± 1.94	2.82×10^{-26}
Tumors: Loss-of-function to benign mutation ratio[2]	0.15± 0.12	0.86± 1.39	1.14×10^{-24}

[1]Essentiality values: 2 – essential; 1- not essential but loss has phenotype; 0 – other

[2]Predicted mutation effects
